# A Comparison of Brain Gene Expression Levels in Domesticated and Wild Animals

**DOI:** 10.1371/journal.pgen.1002962

**Published:** 2012-09-27

**Authors:** Frank W. Albert, Mehmet Somel, Miguel Carneiro, Ayinuer Aximu-Petri, Michel Halbwax, Olaf Thalmann, Jose A. Blanco-Aguiar, Irina Z. Plyusnina, Lyudmila Trut, Rafael Villafuerte, Nuno Ferrand, Sylvia Kaiser, Per Jensen, Svante Pääbo

**Affiliations:** 1 Department of Evolutionary Genetics, Max Planck Institute for Evolutionary Anthropology, Leipzig, Germany; 2 Lewis Sigler Institute for Integrative Genomics, Princeton University, Princeton, New Jersey, United States of America; 3 CAS–MPG Partner Institute for Computational Biology SIBS, Shanghai, China; 4 CIBIO, Centro de Investigação em Biodiversidade e Recursos Genéticos, Vairão, Portugal; 5 Departamento de Zoologia e Antropologia–Faculdade de Ciências da Universidade do Porto, Porto, Portugal; 6 Fernan Vaz Gorilla Project, Port-Gentil, Gabon; 7 Department of Biology, University of Turku, Turku, Finland; 8 Instituto de Investigación en Recursos Cinegéticos, IREC (CSIC, UCLM, JCCM), Ciudad Real, Spain; 9 Institute of Cytology and Genetics, Siberian Branch of the Russian Academy of Sciences, Novosibirsk, Russia; 10 Department of Behavioural Biology, University of Münster, Münster, Germany; 11 IFM Biology, Division of Zoology, Avian Behaviour Genomics and Physiology Group, Linköping University, Linköping, Sweden; University of Washington, United States of America

## Abstract

Domestication has led to similar changes in morphology and behavior in several animal species, raising the question whether similarities between different domestication events also exist at the molecular level. We used mRNA sequencing to analyze genome-wide gene expression patterns in brain frontal cortex in three pairs of domesticated and wild species (dogs and wolves, pigs and wild boars, and domesticated and wild rabbits). We compared the expression differences with those between domesticated guinea pigs and a distant wild relative (*Cavia aperea*) as well as between two lines of rats selected for tameness or aggression towards humans. There were few gene expression differences between domesticated and wild dogs, pigs, and rabbits (30–75 genes (less than 1%) of expressed genes were differentially expressed), while guinea pigs and *C. aperea* differed more strongly. Almost no overlap was found between the genes with differential expression in the different domestication events. In addition, joint analyses of all domesticated and wild samples provided only suggestive evidence for the existence of a small group of genes that changed their expression in a similar fashion in different domesticated species. The most extreme of these shared expression changes include up-regulation in domesticates of *SOX6* and *PROM1*, two modulators of brain development. There was almost no overlap between gene expression in domesticated animals and the tame and aggressive rats. However, two of the genes with the strongest expression differences between the rats (*DLL3* and *DHDH*) were located in a genomic region associated with tameness and aggression, suggesting a role in influencing tameness. In summary, the majority of brain gene expression changes in domesticated animals are specific to the given domestication event, suggesting that the causative variants of behavioral domestication traits may likewise be different.

## Author Summary

Over the millennia, humans have turned a range of wild animal species into what we today know as domesticated animals. Domestication has greatly influenced human history and evolution. The changes in the animals are even more drastic. Domesticated animals differ from their wild relatives in appearance, physiology, and behavior. Although these differences are mostly genetically encoded, little is known about which genes contribute to these domestication traits. Changes in gene expression have long been proposed to lead to phenotypic changes in evolution. In this work, we therefore compared gene expression in brains of dogs and wolves, pigs and boars, and domesticated and wild rabbits and guinea pigs. For each of the four domesticated species, we identify gene expression differences that could correlate with behavioral differences compared to wild animals. The majority of expression differences are unique to each domestication event, suggesting that domestication has proceeded through different genetic routes in different species.

## Introduction

**Figure 1:**
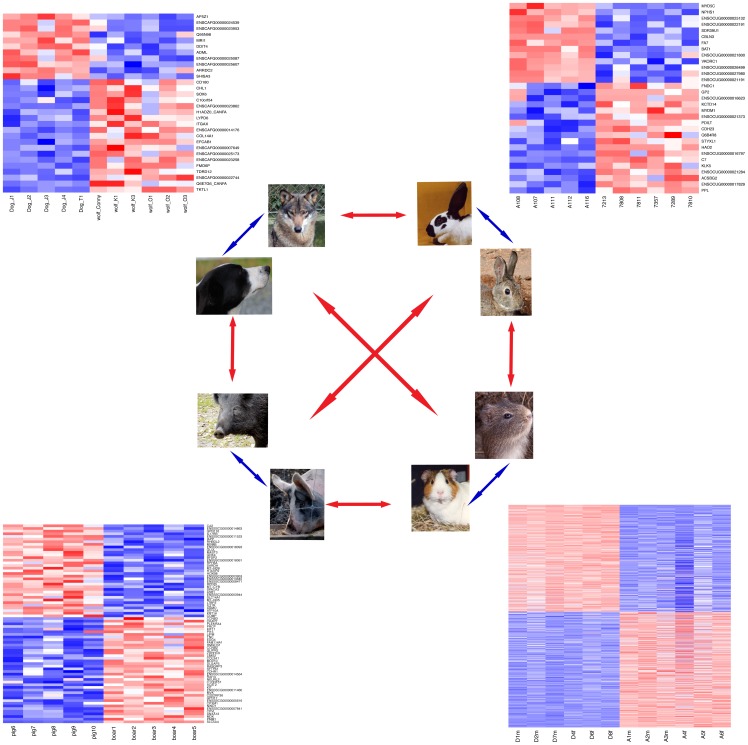
Gene expression in domesticated and wild animals. Cortical gene expression in four domesticated animal species is compared to their wild relatives (blue arrows), followed by comparisons between the four domestication events (red arrows). Next to each domesticated/wild pair, a heatmap shows expression levels of all respective DE genes (Table 1). Genes were individually normalized and sorted by DE p-value, separately for genes up- and downregulated in domestication. Red (blue): lower (higher) expression. Due to the high number of DE genes, gene names are omitted for the guinea pig comparison. See Dataset S2 for details on the DE genes.

Domesticated animals differ from their wild relatives in a number of traits, several of which are shared among different domesticated species [1]. Shared traits include conspicuous coat color variation (Figure 1), reduced cranial volume and skeletal gracilization [2] as well as behavioral traits such as reduced fear, higher levels of adult play, and less aggressive behavior [2], [3]. The genetic basis for most domestication traits is unknown [4], [5], with the exception of genetic variants causing between-breed differences in coat color [6]–[8] and some other breed-specific morphological and physiological traits [9], [10]. In addition, a recent genome-wide survey for positive selection in chicken identified genes that may be involved in domestication-related traits [11]. No genetic variants are known to cause domestication-specific behaviors.

Some of the known genetic variants causing domestication-related phenotypes occur in the same genes or in genes acting in the same physiological pathway in several independently domesticated species. This is particularly true for coat color variation in dogs [6], pigs [7], horses [12] and chicken [8], which is often influenced by variants in the agouti–melanocortin 1 receptor (MC1R) pathway. In addition to domesticated animals, parallel genetic changes leading to similar phenotypic outcomes have been found in *C. elegans* [13], stickleback fish [14], [15], mice [16] and other species [17], suggesting that the genetic basis of phenotypic evolutionary change is to some extent predictable [18].

Genetic changes causing differences in gene expression have long been speculated to underlie phenotypic differences between species [19]. Supporting this notion, there is now a substantial and growing list of changes in *cis*-regulatory DNA that cause differences between species (e.g. [14], [20], [21], reviewed in [17], [18]). Gene expression differences between domesticated and wild animals could therefore be important contributors to domestication-specific features.

As a first step towards identifying such changes, we set out to characterize gene expression in four pairs of domesticated and wild mammals: dogs and wolves, pigs and boars, domesticated and wild rabbits, and domesticated guinea pigs and a relatively distant ancestor (*Cavia aperea*), making use of recent advances in transcriptome sequencing (“RNA-seq” [22], [23]). Because our interest is primarily in behavioral aspects of domestication, we analyzed mRNA levels in the brain. We also study brain gene expression in two lines of rats that have been selected for more than 60 generations for tame and aggressive behavior towards humans [24]. The two rat lines were initiated in 1972 from one population of wild caught rats in order to model the first steps in animal domestication [25], raising the question whether gene expression differences in their brain resemble those in domesticated animals.

## Results

### Gene expression differences between domesticated and wild animals

We obtained tissues from three mammalian domesticated species as well as their corresponding wild ancestral species: dogs and wolves, pigs and wild boars, and domesticated and wild rabbits (Figure 1, Dataset S1 for detailed sample information). These species represent most of the mammalian species for which high-quality tissues and RNA can be obtained from both domesticated animals and their close wild relatives. We isolated mRNA from brain frontal cortex and performed transcriptome sequencing in 5–6 individuals from each of the species. Frontal cortex was chosen because it is involved in many cognitive functions including social behavior [26], [27] and because it can be easily and rapidly identified and dissected in all animals studied. After alignment and quality filtering (Materials and Methods), 4.5–22 million sequencing reads were available per sample (median = 14 million; see Dataset S1 for read counts for each sample). We restricted our analyses to genes that had counts in at least half the samples per comparison, resulting in 15,522–19,306 analyzed genes per species pair (Table 1).

**Table 1:** Pairwise differential expression.

Comparison	Expressed genes	DE genes (fraction/p-value^3^)	Variance^1^ (p-value^3^)	fold change^2^ (p-value^3^)
Dog/Wolf	19,228	30 (0.2%/0.007)	17.7% (0.05)	32% (0.03)
Pig	15,522	75 (0.5%/0.04)	15.3% (0.2)	24% (0.1)
Rabbit	17,262	31 (0.2%/0.02)	12.0% (0.4)	25% (0.3)
Guinea pig	16,755	1,513 (9.0%/0.004)	25.9% (0.004)	40% (0.002)
Rat	19,306	28 (0.1%/0.01)	8.8% (0.5)	20% (0.4)

1 variance explained by domestication/species, mean across expressed genes.

2 absolute fold change, mean across expressed genes.

3 Fraction of all possible permutations of the domestication factor where the respective statistic is matched or exceeded.

**Figure 2:**
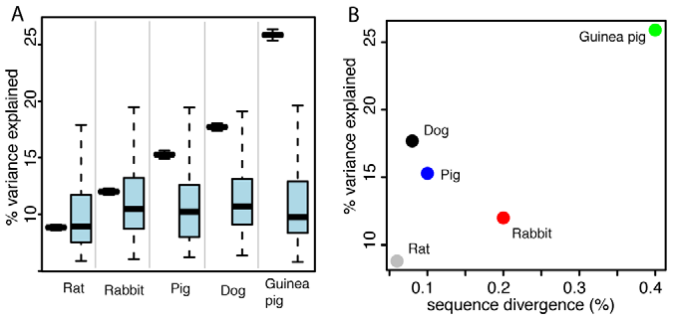
Expression differences between domesticated and wild animals. A. Pairwise expression differences. Plotted for each comparison is in black on the left the mean variance explained by domestication, along with 95% confidence intervals (box whiskers) based on 10,000 bootstraps across genes. On the right in light blue is the null distribution of mean variance explained by domestication based on permutations of the domestication factor (box whiskers comprise 95% of the distribution, central horizontal bar is the median). B. For each comparison, the mean variance explained by domestication across genes is plotted as a function of sequence divergence expressed as the median fraction of nucleotides that differ between any two domesticated and wild animals in that comparison.

We first compared gene expression for each pair of domesticated and wild species separately. On average, the difference between domesticated and wild animals explained 12–18% of gene expression variance in the three species pairs (Figure 2A, Table 1), in agreement with an earlier microarray study on dogs and wolves [28]. However, permutation tests showed that, with the exception of dogs and wolves (p = 0.05), these average differences were not larger than expected by chance (pig: p = 0.2, rabbit: p = 0.4, Table 1). The magnitude of differences is illustrated in Figure 2A, where for each pair the mean variance explained by domestication is plotted next to the null distribution obtained from the random permutations. For dogs, the observed mean is at the 5% tail of the empirical null distribution, while for rabbits and pigs, the observed mean is well inside the null distribution. In line with these small differences, the domesticated and wild individuals only weakly clustered in principal component analyses (PCA) when analyzing all expressed genes (Figure S1). At a 10% false discovery rate (FDR, Benjamini-Hochberg [29] corrected, estimated separately for each pair), 30, 75, and 31 genes were found to be significantly differentially expressed in dogs, pigs, and rabbits when compared to their wild relatives (“DE genes”, Figure 1 and Table 1, see Dataset S2 for a complete list). In all three species pairs, these numbers of DE genes were significantly larger than those found in permutations (dogs: p = 0.007, pigs: p = 0.039, rabbits: p = 0.024). Thus, cortical transcriptomes differ modestly between domesticated and wild species, with a few dozen of differentially expressed genes.

### Gene expression and DNA sequence divergence

To put the amount of gene expression difference between domesticated animals and their wild relatives into perspective, we compared them to gene expression differences between other pairs of animals at different evolutionary distances from each other. We gathered RNA-seq data from frontal cortex from two lines of rats that have been selected for more than 60 generations for tame and aggressive behavior towards humans, respectively [24], as well as from domesticated guinea pigs (*Cavia porcellus*) and from *Cavia aperea*, a wild cavy that is a distant relative of domesticated guinea pigs [30] (Figure S1). The overall extent of expression differences between dogs and wolves, pigs and wild boars, and domesticated and wild rabbits were of intermediate magnitude to those between the two rat lines and the two cavy subspecies (Figure 2A, Table 1, see Dataset S2 for a list of DE genes in guinea pigs and rats).

To determine to what extent these gene expression differences correlate with DNA sequence divergence in expressed genes, we searched the RNA-seq data for single nucleotide variants (SNVs). We identified more than 24,000 SNVs per comparison and were able to assign genotypes to all individuals at 4,300–18,000 SNVs (Table 2). In contrast to gene expression levels, PCA of SNV data clearly separated domesticated and wild animals (Figure S2). We used the genotype data to quantify the degree of genetic diversity and divergence in each domesticated/wild pair (Table 2). Nucleotide diversity in domesticated dogs, rabbits and guinea pigs was lower than among the respective wild animals. By contrast, nucleotide diversity was higher in the pigs compared to wild boars.

**Table 2:** Sequence differences.

Species pair	Total SNV positions	Autosomal SNVs w/ complete data	Number (fraction) of fixed differences	Number (fraction) of shared^1^ SNVs	Exonic autosomal bases w/ complete data	Median nucleotide divergence	Median nucleotide diversity (1/1000 bp)^3^
Dog/Wolf	36,768	8,778	115 (1.3%)	4,504 (51.3%)	4,638,464	0.08%	0.26/0.34
Pig/Boar	30,508	9,831	180 (1.8%)	4,534 (46.1%)	4,355,361	0.1%	0.55/0.25
Rabbit	80,681	14,873	133 (0.9%)	6,509 (43.8%)	2,013,023	0.2%	0.51/1.47
Guinea pig	84,269	18,710^1^	10,181 (54.4%)	175 (0.9%)	2,774,323^2^	0.4%	0.13/0.61
Rat	24,065	4,387	260 (5.9%)	1,835 (41.8%)	5,088,072	0.06%	0.13/0.15

1 SNVs where both alleles segregate in both the domesticated and the wild animals.

2 Because the guinea pig genome is not assembled into chromosomes, these data include X-chromosomal sequences.

3 domesticated (tame)/wild (aggressive).

In dogs, pigs, and rabbits, more than 43% of the SNVs segregated in both the domesticated and the wild animals, while less than 2% of the SNVs were fixed differences where all domesticates carry one and all wild animals the other allele. By contrast, the two species of guinea pigs shared only 0.9% of the SNVs while 54% of the SNVs were fixed for different alleles.

Gene expression divergence as measured by the mean fraction of variance explained by domestication was roughly correlated with genetic distance, although this correlation was primarily driven by the large differences in the guinea pigs and the small differences in the rats. The other domesticated/wild comparisons fell in between these extremes (Figure 2B).

### Functional annotation of expression differences

To better understand the potential functional relevance of gene expression differences, we examined the annotated functions of the DE genes in dogs, pigs, and rabbits. The genes with the most significant differences are presented here, while Dataset S2 shows the full lists of DE genes.

The gene with the most significant (p = 5e-24) expression difference between dogs and wolves is transketolase-like 1 (*TKTL1*), with a ∼47-fold higher expression in dogs. *TKTL1* encodes an enzyme that promotes aerobic glycolysis, an inefficient mode of ATP production found in cancer cells [31]–[33]. Conversely, the gene with the most significant (p = 3e-7) expression difference and higher expression in wolves (2.5 fold) is *AP5Z1/KIAA0415*, a proposed subunit of a protein complex involved in trafficking proteins from endosomes to other membrane compartments in the cell [34].

The most significant gene with higher expression in pigs is *SLC5A4*/*SGLT3* (p = 2e-24, 5-fold higher), encoding a membrane-bound sensor of extracellular glucose levels [35]. The most significant gene with higher expression in boars is carbonic anhydrase 2 (*CA2*, p = 2e-8, 2.5-fold higher). The enzyme encoded by this gene is one of several isozymes that catalyze the reversible hydration of carbon dioxide. Mutations in this gene cause osteopetrosis, a metabolic bone disease also leading to cerebral calcification [36]. In addition, the gene *KIT* had 2-fold higher expression in pigs compared to boars (p = 5e-5). *KIT* is a tyrosine-kinase receptor involved in melanoblast migration [37]. Variation in *KIT* has been linked to white spotting in numerous domesticated species [38]–[41] as well as in the tame rats [25].

In rabbits, the most significant gene with higher expression in domesticates was periplakin (*PPL*), a cytoskeleton-associated protein that plays a role in cell-cell-adhesion [42]. Conversely, the most significant gene with lower expression in domesticated rabbits was myosin 5C (*MYO5C*, p = 2e-19, 4.5-fold lower), a molecular motor protein that plays a role in trafficking secretory granules [43].

We next examined the biological functions of DE genes as identified in the Gene Ontology (GO) database. To obtain a broad overview of the biological functions that differ in expression between dogs, pigs, rabbits and rats, we tested all genes with a nominal difference (p<0.05) for GO enrichment in these species. Due to the large number of expression differences in guinea pigs, we tested genes at a 10% FDR in this species pair. Dataset S3 lists all GO terms that were enriched at a nominal significance of p<0.01. The DE genes clustered in a wide variety of functional categories. For example, genes with higher expression in dogs compared to wolves showed a substantial enrichment of GO terms related to the immune system (e.g. “immune system process”, p = 7e-10; leukocyte migration, p = 4e-5). Immune system terms were also enriched among upregulated genes in pigs (e.g. “immune system process”, p = 0.0006), guinea pigs (“immune system process”, p = 0.005) and tame rats (e.g. “immune response”, p = 0.006) but at lower significance than in dogs. No immune system terms were enriched among rabbits.

To assess if the expression changes between domesticated and wild animals may have been driven by positive selection at local regulatory sites during domestication, we compared them to genome maps of positive selection. For dogs, we compared to selected regions published by von Holdt *et al.* [44], whereas we used unpublished selection maps for domesticated pigs (C. Rubin and L. Andersson, personal communication) and rabbits (M. Carneiro, unpublished). For dogs, pigs and rabbits, none, none and one (*FNDC1*) of the respective DE genes overlapped with regions under positive selection during domestication.

### Comparison of expression differences between domesticated species

Next, we examined whether the same genes have changed their expression in different domesticated species. First, we asked if the DE genes in one species pair are also DE in another species pair. In these analyses, we considered only expressed 1∶1 orthologues between the given pairs (Table S1 shows the numbers of genes that were analyzed in each comparison). Twelve genes were shared between guinea pigs and pigs (Fisher's exact test (FET) p = 0.003). One gene each was DE in guinea pigs and either dogs or rabbits (p = 0.8). Dogs, pigs and rabbits shared no DE genes (Table 3, above diagonal). This first test is strict on the one hand, as it requires genome-wide significance in both respective species pairs that are compared. It may therefore miss instances where a gene narrowly fails to reach significance in one of the species. On the other hand, the test is permissive in that it is blind to the direction of expression change – a gene may be DE in both comparisons but be higher in domesticates in one and lower in domesticates in the other species. Therefore, we next asked if the direction of DE genes in one species pair is predictive of the direction of expression difference in another species pair, irrespective of significance in the second pair (*e.g.* Do the genes that are DE and higher in dogs compared to wolves have higher expression in pigs compared to boars?). There was no comparison where differential expression in one species predicted expression change in another species pair (Table 3, below diagonal).

**Table 3:** Pairwise overlap of DE genes among domestication events.

	Dog	Pig	Rabbit	Guinea pig	Rat
Dog	–	No overlap	No overlap	n = 1, p = 0.8	No overlap
Pig	2.1, p = 0.2	–	No overlap	n = 12, p = 0.003	No overlap
Rabbit	1.0, p = 0.6	1.2, p = 0.4	–	n = 1, p = 0.8	n = 1, p = 0.02
Guinea pig	1.4, p = 0.4	1.1, p = 0.3	0.9, p = 0.8	–	n = 5, p = 0.006
Rat	1.1, p = 0.5	1.5, p = 0.4	0.9, p = 0.7	0.9, p = 0.9	–

Above diagonal: number of genes that are DE in both species, with Fisher's exact test p-values.

Below diagonal: Odds ratios of FET testing if DE genes in the one species predict expression direction in the other species, with Fisher's exact test p-values. Only the more significant direction is shown (see Materials and Methods for details).

As a third approach to test for overlap among the different domestication events, we correlated median expression differences across all expressed orthologues between two species pairs. The correlation coefficients were positive in all cases (Table 4). However, with the exception of dogs and guinea pigs (spearman's rho = 0.3, permutation p = 0.033), the strength of each pairwise correlation did not exceed that in 1,000 permutations of the domestication factor (Table 4).

**Table 4:** Pairwise correlations between expression differences among domestication events.

	Dog	Pig	Rabbit	Guinea pig	Rat
Dog	–				
Pig	0.2, p = 0.13	–			
Rabbit	0.13, p = 0.3	0.08, p = 0.3	–		
Guinea pig	0.3, p = 0.033	0.14, p = 0.16	0.06, p = 0.3	–	
Rat	0.15, p = 0.3	0.08, p = 0.3	0.03, p = 0.4	0.09, p = 0.3	–

Shown are correlations (Spearman's rho) of median expression differences between domesticated and wild animals across all expressed orthologues, with permutation-based p-values.

### Global analyses of gene expression among domesticated and wild animals

Added statistical power to detect expression similarities among different domestication events can be gained by combining all the domesticated and wild samples into one global analysis of variance (ANOVA). We initially focused on 6,901 1∶1 orthologues with reliable expression in dogs, wolves, pigs, boars and domesticated and wild rabbits, using variance-stabilized data [45]. Across genes, a median of 70% of the variance was explained by differences between species pairs (permutation-based p<0.001, Table 5), sex had no discernable effect (1%, permutation p = 0.9), while domestication explained 4.3%, a nearly significant effect (permutation p = 0.051). Similar results were obtained from independently derived gene expression measures in units of FPKM (fragments per kilobase of gene model and million reads, [22]) (Table 5); here the fraction of variance attributable to domestication (3.2%) was significant (permutation p = 0.040).

**Table 5:** Gene expression across domesticated animals.

Data type	Model	Species pairs in analysis	% median variance (p-value)^1^			domestication-related genes^2^
			Species pair	Sex	Domestication	
VSD	Linear	Dog, pig, rabbit	70 (<0.001)	1.1 (0.9)	4.3 (0.051)	709
FPKM	Linear	Dog, pig, rabbit	68 (<0.001)	2.0 (0.4)	3.2 (0.040)	587
count	Negative binomial	Dog, pig, rabbit	–	–	–	551
VSD	Linear	Dog, pig, rabbit, guinea pig	75 (<0.001)	0.7 (0.95)	5.1 (0.006)	920
FPKM	Linear	Dog, pig, rabbit, guinea pig	71 (<0.001)	1.1 (0.7)	3.7 (<0.001)	667
count	Negative binomial	Dog, pig, rabbit, guinea pig	–	–	–	711

1 p-values are the fraction of 1,000 random permutations of the corresponding factor where the observed statistic is matched or exceeded.

2 number of genes with nominally significant (per-gene ANOVA p<0.05) effect for the domestication factor and with the same direction of expression change in the domesticated species. VSD: variance-stabilized data.

**Figure 3:**
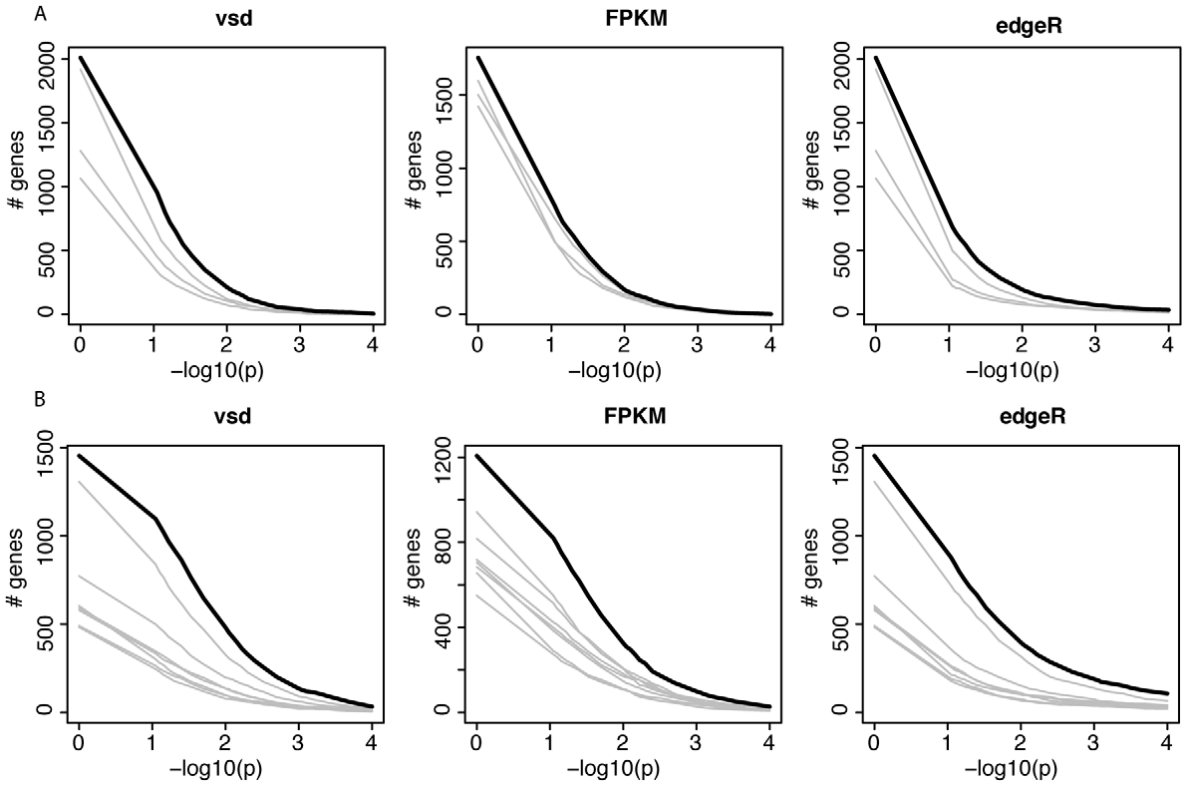
Gene expression across domesticated species. A and B) For each of three models, the figure shows the number of genes that match or exceed the p-value for the domestication factor and that are expressed in the same direction in domesticated animals. The thick black line shows the real data. Each grey line shows the result from one of the possible extreme permutations (see Note S1 for details). p = 1 is included to show the effect of only requiring genes to be expressed in the same direction, irrespective of significance. vsd: variance stabilized data. A) joint analyses of dogs, pigs and rabbits. B) As in A, but including guinea pigs.

We next sought to identify individual “domestication-related genes” that meet two criteria: 1) a significant effect of domestication in the combined ANOVAs of dogs, wolves, pigs, boars and domesticated and wild rabbits; and 2) expression differences in the same direction between the respective domesticated and wild species. At a nominal significance cutoff of p<0.05, hundreds of such genes were found (Table 5). However, because thousands of genes are analyzed, some fraction of genes is expected to meet the above criteria simply by chance. Therefore, random permutations are typically used to estimate the false discovery rate (FDR). A complication arises in estimating FDR for the apparent domestication-related genes. Most random permutations of the domestication factor reduce the expression differences between domesticated and wild animals within each species pair that are present in the real data. For the across-pair domestication p-value, random permutations are therefore biased towards larger p-values, resulting in an anticonservative test. The complication can in principle be overcome by only using “extreme” permutations where all domesticated and wild animals within a species pair are exchanged. The magnitude of within-pair differences then remains intact, while only their direction is permuted relative to the other species pairs. Note S1 and Figure S3 provide a detailed discussion of this effect. With three species pairs, three extreme permutations are possible. Figure 3A and Figure S4A show that more genes reached significance in the real data at all tested p-value thresholds than in the other three permutations, although to a small degree. Similar results were obtained from three statistical models based on two independent measures of gene expression (Figure 3A and Figure S4B and S4C), although in the FPKM data, the excess of lower p-values in the real data was very slight and did not extend into the most extreme tail of the p-value distribution (Figure S4B).

To add statistical power to the joint analyses of dogs, pigs and rabbits, we turned to *C. porcellus* and *C. aperea*. The divergence between *C. porcellus* and *C. aperea* is mainly due to an older pre-domestication population split [30], but domestication of *C. porcellus* is likely to have caused additional expression differences. We asked if apparent domestication-related genes from the joint dog, pig and rabbit analyses predict expression change in domesticated guinea pigs. We selected the 209 genes that had nominally significant (p<0.05) p-values for the domestication factor in the three models in the joint analyses of dogs, pigs and rabbits, and whose expression differs from wild animals in the same direction in these three domesticated species. At 150 of the 202 orthologues that were expressed in guinea pigs, *C. porcellus* differed from *C. aperea* in the same direction as the other domesticated animals differed from their respective wild relatives (one-sided FET: odds ratio = 11.4, p = 7e-14). This degree of overlap was stronger than in gene sets of the same size derived from the three possible extreme permutations (odds ratios = 0.6, 2.7 and 3.0). Similar expression of these 150 genes in domesticated compared to wild animals (see Dataset S4 for a complete list) was not an artifact caused by transcript sequence differences between wild animals and the reference genomes (Note S2 and Figure S5). None of the 150 genes overlapped with regions for evidence of positive selection during domestication in dogs [44], and one (*LYN*) overlapped with a positively selected region in domesticated pigs (C. Rubin and L. Andersson, personal communication). While five genes (*AGPS*, *ALS2CR8*, *C1orf27*, *C2orf77*, *MYO1B*) overlapped with positively selected regions in domesticated rabbits (M. Carneiro, unpublished), this was not more than expected by chance (one-sided FET, odds ratio = 1.1, p = 0.8).

We then added the cavies to the ANOVA analyses to increase the possible number of extreme permutations where all members of a domestic/wild pair can be switched. As expected given the large differences between the two cavy species, their addition increased the magnitude and significance of the fraction of variance explained by domestication (Table 5). Adding a fourth pair doubles the number of possible extreme permutations to seven. The real data produced more domestication-related genes than these permutations, but again to a small degree (Figure 3B and Figure S6).

**Figure 4:**
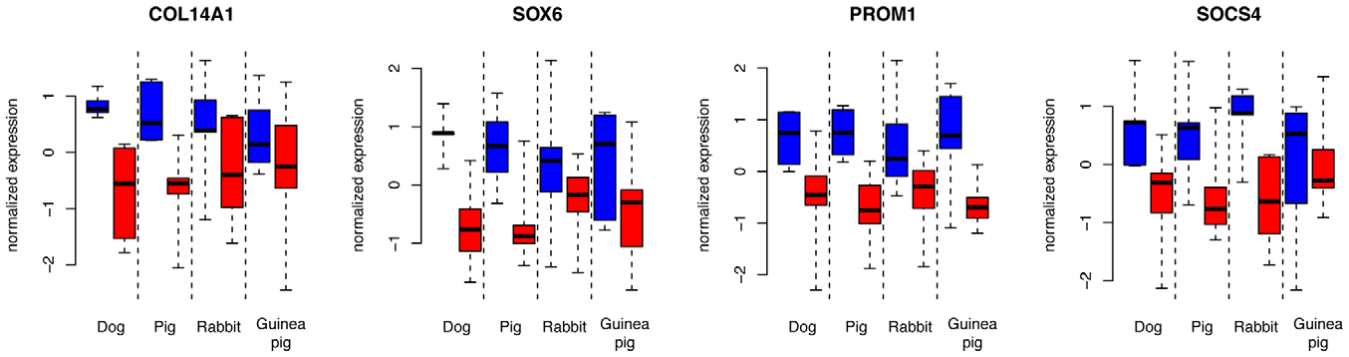
Expression levels of four genes with common expression in domesticated dogs, pigs, rabbits, and guinea pigs. Blue: domesticated animals, red: wild animals. Shown are the four genes with the lowest p-values for the domestication factor across dogs, pigs and rabbits, and with expression change in the same direction in these three species as well as guinea pigs. Expression levels are from variance stabilized data, separately normalized to the median in each species pair.

In sum, there is suggestive and tentative evidence for some degree of shared gene expression differences associated with domestication in these four domesticated species. Figure 4 shows the four most extreme genes with shared expression among domesticated dogs, pigs, rabbits and guinea pigs. These genes include the genes *SOX6* (SRY (sex determining region Y)-box 6) and *PROM1* (prominin 1/CD133). The transcription factor *SOX6* is a modulator of cell fate during neocortex development [46]. *PROM1* is involved in neuronal stem cell maintenance [47], and its expression is negatively correlated with adult neurogenesis in mice [48]. In the individual comparisons, dogs compared to wolves had significantly higher expression of *SOX6* (Figure 1 and Dataset S2).

### Expression differences between tame and aggressive rats

**Figure 5:**
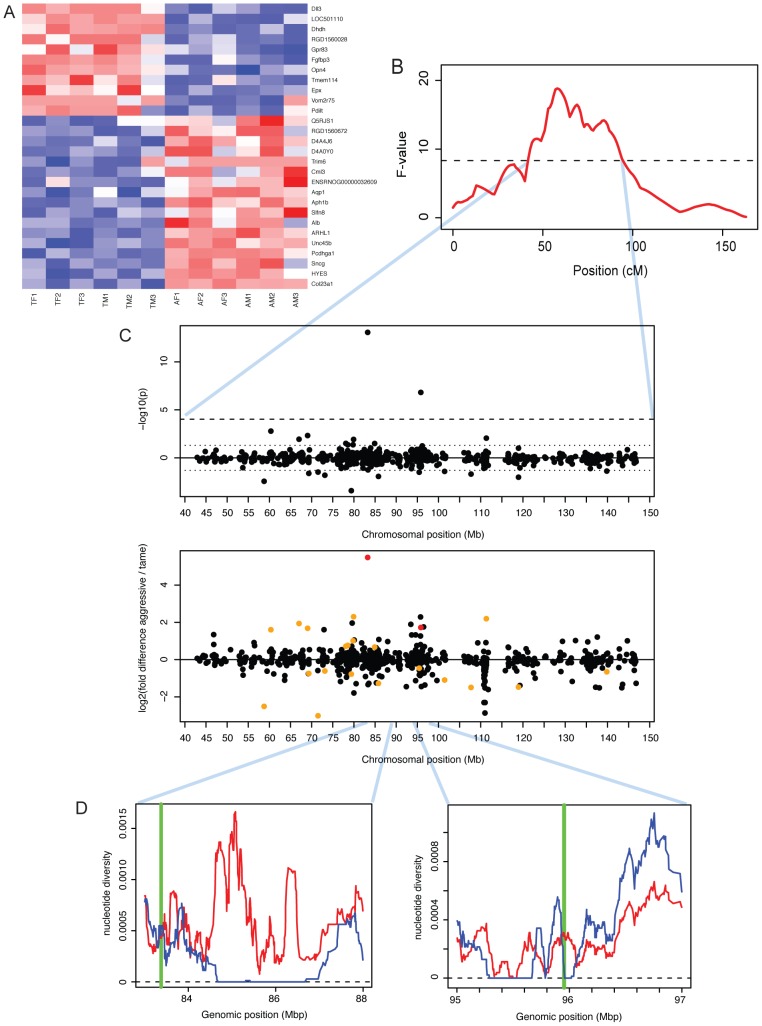
Expression differences between tame and aggressive rats. A. Heatmap showing expression levels of DE genes. Genes were individually normalized and sorted by DE p-value, separately for genes up- and downregulated in domestication. Red (blue): lower (higher) expression. B. A QTL for tameness is located on chromosome one [25]. The x-axis shows the genetic position along chromosome one in centiMorgan (cM). The F-value is a measure of the likelihood of the presence of a QTL. The dashed horizontal line is the genome-wide significance threshold. See [25] for details. C. Expression differences in the tameness QTL region. Top panel: significance and location for each gene. P-values were signed so that positive (negative) values correspond to genes with higher expression in aggressive (tame) rats. Dashed lines: genome-wide 10% FDR threshold, dotted lines: p = 0.05. Lower panel: fold changes. Red: genes with FDR<10%, orange: genes with p<0.05. D: positions of *Dll3* and *Dhdh* (green vertical lines) compared to patterns of DNA polymorphism in the founder animals of the QTL pedigree used to identify the tameness QTL [49]. Blue (red) line: nucleotide diversity in the tame (aggressive) founder animals.

The tame rat selection line was conceived as an experimental model of the early steps of animal domestication [25]. The rats had been selected for more than 60 generations for the absence of aggression towards humans, and today are extremely tolerant of human presence and handling. By contrast, rats from a parallel selection line for aggression towards humans respond with fierce attacks to any attempts of handling [24]. At a 10% FDR, 28 genes were differentially expressed between the tame and the aggressive rats (Figure 5A). We asked whether the selection for tameness has led to gene expression changes analogous to those in domesticated animals. The rats shared one DE gene with rabbits (*PDILT*), but no additional significant overlap between the rat comparison and the domesticated and wild comparisons was observed (Table 3; FET test for shared direction with 209 domestication-related genes from dogs, pigs and rabbit: p = 0.06).

In previous work, we have used a cross between the tame and the aggressive rats to identify two regions of the genome that influence tameness and aggression (Figure 5B) [25]. These quantitative trait loci (QTL) are large and contain many genes. Genes with expression differences in the brain in these QTL may contribute to tameness. A major tameness QTL on chromosome one contained two DE genes (Figure 5C): delta-like protein 3 (*DLL3*) is the most significant DE gene with higher expression in the aggressive rats, while dihydrodiol dehydrogenase (dimeric) (*DHDH*) is the third-most significant of these genes. *DLL3* in particular has drastically lower expression in the tame rats, corresponding to a 45-fold expression difference. We next asked whether these two genes are located in regions of the QTL that may have experienced positive selection during the artificial selection for tameness and aggression. For this comparison, we used published DNA sequence data from all exons in the QTL region from the four tame and the four aggressive founder animals of the pedigree used to identify the QTL [49]. *DLL3* is close to a region where the tame, but not the aggressive founders have no sequence diversity (Figure 5D). *DHDH* is directly on the border of a similar region. Both genes are located close to the center of the QTL region. Among the genes in the second tameness QTL that had earlier been found on chromosome eight [25], none were DE.

In addition, the gene synuclein gamma (*SNCG*) had ∼4 fold higher expression in tame than in aggressive rats, but is not localized in any of the tameness QTL. However, *SNCG* was found to be higher expressed in the cortex of dominant than in those of submissive rats, suggesting a possible link to aggressive behavior [50].

## Discussion

We compared gene expression levels in the brains of three pairs of domesticated species and their wild ancestor species. Gene expression differences between domesticated animals and their close wild ancestors were overall small in magnitude, and only a few dozen genes were differentially expressed. We aimed to put these expression differences in context by comparing them to those between domesticated guinea pigs and a more distantly related wild species of cavy and to two lines of rats that were selected for tame and aggressive behavior towards humans as a model for animal domestication [25]. The dog, pig and rabbit comparisons fell in between these two extremes, as expected given most gene expression change accumulates over evolutionary time in parallel with sequence divergence [51]–[52]. The most parsimonious explanation for this correlation is that most observed gene expression differences between species are selectively neutral or nearly neutral [53] or constrained by negative selection [54]. Most of the gene expression changes between domesticated and wild animals are therefore unlikely to have been individually positively selected. In line with this view, expression changes in dogs, pigs and rabbits did not significantly overlap with regions under positive selection associated with domestication, suggesting that the expression changes were not individually driven by positive selection at *cis*-regulatory sites. The expression changes may be caused by genetic differences in *trans*, neutral differences in *cis*, and/or physiological correlates of domestication. We suggest that at present, the differentially expressed genes are best described as domestication phenotypes, i.e. consequences rather than cause of other domestication traits.

The correlation between expression divergence and evolutionary distance was further corroborated by the sequence variation data we extracted from the RNASeq data, an increasingly common strategy [55]–[60]. For this paper, we have chosen to focus our sequence analyses on genome-wide summary statistics (Table 2). More detailed analyses of these sequence data are reported elsewhere ([60] and Carneiro, Albert *et al.*, submitted). For all comparisons but the distantly related guinea pigs, there was allele sharing at more than 41% of the identified SNVs, illustrating the very recent population splits between domesticated and wild animals. The sequence data also showed reduced genetic variation in domesticated compared to wild dogs, rabbits and guinea pigs, as expected from domestication-associated bottlenecks [61]–[63]. By contrast, the pigs had higher genetic diversity than the boars (Table 2). While counterintuitive at first glance, this result is consistent with the literature [64]. Specifically, the domesticated pigs we sampled derived from a three-breed cross population from breeds Hampshire×Yorkshire×Swedish Landrace. Some European domesticated pigs (including Landrace [65]) derive from historical admixture between Asian and European pigs [66]. In addition, European wild boars have lower genetic diversity than Asian wild boars [67], possibly due to historical population bottlenecks [65]. Therefore, the hybrid origin of our domesticated pig samples explains their relatively high nucleotide diversity. Finally, the sequence data clearly show that *C. aperea* is too divergent from domesticated guinea pigs to be the direct wild ancestor. This finding supports earlier studies of the mitochondrial *cytochrome b* gene in cavies [30], [68], while being based on a much larger set of genetic markers across the entire genome.

The domesticated species we analyzed are separated from each other by millions of years of evolution and have been domesticated at different times, on different continents, and probably for different purposes. Today, dogs are primarily kept as companion or working animals, pigs for meat production, while rabbits and guinea pigs serve both purposes. These different uses likely entailed different selection pressures acting on each of these domesticated species. At the same time, all domesticated animals, including those sampled here, share a range of similarities. These include variation in coat color and morphology, reduced cranial capacity, and gracile skeletons as well as a suite of behavioral traits including reduced aggression and tolerance of human presence. We therefore examined whether parallel patterns in brain gene expression exist among different domesticated species.

The results show that on a gene-by-gene level, the overlap of genes that differ between the species pairs is minimal, and for the most part not predictive of the direction of expression change. The correlations between the gene expression differences across all genes were, although always positive, not stronger than expected by chance (with the exception of dogs and guinea pigs where there were marginal similarities). In the joint ANOVA, less than 5% of gene expression variance across genes was explained by domestication. Because all comparisons between species pairs were performed exclusively on 1∶1 orthologues (Materials and Methods), the results are restricted to genes that have not experienced gene duplication events during the long evolutionary timescales that separate the domesticated species studied here. If domestication has similarly affected the expression of a gene in two species where the ancestral gene was duplicated along one or both lineages, we would miss this instance of sharing. Similarly, we relied on publicly available gene annotation that very likely is of variable quality in the species we studied. Some real instances of sharing between domesticated animals may be missed if the corresponding gene is wrongly or not at all annotated in one or both species.

Two observations prevent a complete rejection of shared gene expression differences among domesticates. First, apparent “domestication-related” genes identified among dogs, pigs and rabbits did significantly predict the direction of expression differences between domesticated guinea pigs and *C. aperea*. Second, more genes reached significant domestication p-values in the actual data than in extreme permutations where only the direction of the differences in each species pair is switched (Figure 3). Because only few extreme permutations are possible with the four pairs studied here, this result does not constitute a stringent significance test. Unfortunately, adding more pairs is currently not practical since high-quality tissue samples from close wild relatives of many domesticated animals are difficult or impossible to obtain because the wild animals are extinct, endangered, and/or live in remote areas. From the presently available data, we conclude that there is at most suggestive evidence for shared expression differences.

Nevertheless, the potential role of expression change at genes with the strongest evidence for sharing appears interesting. For example, the genes *SOX6* and *PROM1* have different expression in the same direction in all four domesticated species (Figure 4). Both genes play roles in brain development, suggesting that their altered expression in domesticates may reflect differences in the development or maturation of the frontal cortex in domesticated animals. This possibility is especially intriguing since one feature of animal domestication is “neoteny”, the retention of juvenile traits and behaviors into adulthood [2], [3]. The altered expression of development-related genes may reflect anatomical differences between domesticated and wild animals that are in turn due to delayed or arrested cortical development in domesticated animals. It would be interesting to study how brain anatomy and gene expression develop during ontogeny in domesticated and wild species.

So far, we considered the hypothesis that the expression levels of a common set of genes have changed in animal domestication. There is a possibility that expression change at different genes may still affect the same or similar biological pathways or functions. However, the DE genes in the different domestication events were enriched for a variety of functional categories that did not show obvious overlap among the species comparisons. One possible exception are functional categories related to the immune system, which showed enrichment in genes with higher expression in dogs, pigs, guinea pigs and the tame rats. As the individual animals used here were matched for environment wherever possible, it is unlikely that these similarities are simply due to similar exposure to infectious agents in these individuals. Genes involved in the immune system generally evolve rapidly [69], making the immune system a likely target of modification during the relatively short time span of domestication. In addition, animal domestication entailed shifts towards more crowded living conditions in herds and/or increased exposure to humans and other domesticated species [1]. Domesticated animal species are therefore likely to have become exposed to particularly strong selection pressures on their immune systems, perhaps resulting in elevated expression of some immune-system genes.

In spite of the lack of strong overlap between domesticated events, some of the genes that differ in just one of the pairs were interesting. For example, the *KIT* gene was more highly expressed in pigs than in boars. Genetic variants in *KIT* cause coat color differences in a wide range of species, including domesticated pigs [41], horses [38] and cattle [39]. Differential expression of this coat color locus in the brain is noteworthy because genetic variants that influence coat color are sometimes thought to also influence aggression [70]–[72], leading to the idea that selection for tameness may have caused the rich variation in coat color in domesticated animals as a side effect [2]. Although such a genetic link between tameness and white coat spotting was not found in the tame and the aggressive rats [25], there remains a possibility that coat color and behavioral differences between pigs and boars may be connected via differential expression of the *KIT* gene.

We identified 28 genes that differ in expression between the tame and the aggressive rats. The rats have been selected for more than 60 generations for their behavioral response towards humans, with the goal of modeling the early steps of animal domestication in an experimentally controlled fashion [25]. Today, the rats differ drastically in their behavior. Tame rats show no fear towards humans and can easily be handled, while the aggressive rats vigorously attack humans at any attempts of handling. In the context of the present study, the tame rats are therefore of particular interest as they provide a model where only one of the traits that characterize domesticated animals was selected, and where environmental influences were strictly controlled both in the actual animals used in the study and during the entire course of selection. The only DE gene that overlapped between the rats and any domesticated species is *PDILT*, which encodes an enzyme that acts as a chaperone in the endoplasmatic reticulum in testis [73]. Its function in the brain is not known. Further, *PDILT* has lower expression in the tame rats than the aggressive rats, but higher expression in domesticated than in wild rabbits (Figure 1 and Dataset S2), whereas an expression difference causing tameness in the rats and rabbits would be expected to be in the same direction. The nearly complete absence of overlap between the genes that differ in expression between domesticated and wild animals and the tame and aggressive rats suggests that a simple universal relationship between tameness and cortical gene expression does not exist. We can however not rule out that expression differences in brain regions other than cortex (e.g. the thalamus/hypothalamus [28], [74] or the amygdala [28]) may be more correlated between the rats and domesticated animals, and among other domesticated animals.

Notably, two of the most differentially expressed genes between the tame and the aggressive rats were located in a previously identified QTL that influences tameness and aggression in these rats [25]. *DLL3* is an orthologue of the *Drosophila Delta* gene, the ligand of the Notch protein which is involved in developmental patterning of the embryo [75]. The function of *DLL3* in adult brain is not known. *DHDH* is a member of the family of dihydrodiol dehydrogenases, which catalyze reactions in the metabolism of xenobiotics and sugars [76]. Within the QTL region, both of these two genes were either close to (*DLL3*) or inside (*DHDH*) regions where the tame founder animals of the pedigree used to map the QTL have no sequence variation [49]. This pattern of no sequence variation in tame but not aggressive rats may indicate that these regions could have experienced positive selection for tameness during the artificial selection [49], . Stronger selection in tame than in aggressive rats is consistent with the fact that the tame rats differ more in behavior from wild-caught controls than the aggressive rats [78]. Further, the causative variant at this tameness QTL was estimated to be fixed among the tame founder rats, but to segregate among the aggressive rats [49]. In sum, *DLL3* and *DHDH* are good candidates for influencing tameness and aggression in the rats based on their differential expression in brain and their location in the major tameness QTL in the rats. Future work will need to clarify whether the expression differences at these two genes are heritable, and whether altered expression of these two genes in the brain has functional consequences. Similarly, the gene *SNCG*, while not located in a tameness QTL, was previously linked to aggressive behavior in rats [50]. *SNCG* is a member of the synucleins, a group of proteins that influence dopamine and glutamate release in the brain [50]. If its expression is regulated in *trans* by the tameness QTL, *SNCG* may provide a mechanistic link between the QTL and tameness.

In sum, we identified gene expression differences in frontal cortex in four pairs of domesticated and wild species and between genetically tame and aggressive rats. The respective differences were mostly independent from each other. An important question for future research will be if the genetic variants that cause similar traits in different domestication events (e.g. in behavior or physiology) are similarly species-specific.

## Materials and Methods

### Animals and tissue collection

Brain frontal cortex tissue was obtained from dogs, wolves, domesticated and wild pigs, guinea pigs and rabbits, and from genetically tame and aggressive rats. Unless stated otherwise, all domesticated and wild animals per pair were sacrificed on the same day, in the same facility and using identical procedures, and tissues were extracted immediately after death and frozen in liquid nitrogen or on dry ice. Within each species, all animals had similar ages.

Five unrelated dogs (*Canis familiaris*, two females; one golden retriever, one white terrier, and three dogs of mixed breed origin) were obtained from veterinary practices and animal shelters in Leipzig, Germany. Six unrelated wolves (*Canis lupus*, two females) were obtained from animal parks in Germany and Austria. All canines were old adults and either died of natural causes or were euthananized for medical reasons; they were not killed for the purpose of this study. Three wolf heads and one whole wolf had been frozen at −20°C shortly after death; we dissected brains from the frozen heads without thawing the samples and stored them at −80°C.

European wild boars (*Sus scrofa*) and domesticated pigs (derived from a three-breed cross population from breeds Hampshire×Yorkshire×Swedish Landrace) (age five weeks, five females each) were reared on separate Swedish farms with similar living conditions. The animals were kept with their mother outdoors in large enclosures, and were fed similar commercial pig feed. The sampled domesticated pigs were from separate litters, whereas the wild boars were sampled from one enclosure with five sows and their litters. Within a few days from each other, pigs were killed with a gunshot through the heart from a distance of 10–20 cm while they were feeding. The brain was dissected and snap-frozen in liquid nitrogen within 10 min of killing. The procedure was performed under licence from the regional ethical committee for animal experiments in Linköping, Sweden.

Wild rabbits (*Oryctolagus cuniculus*, six unrelated adult animals each, three females each) reared in outdoor enclosures and domesticated rabbits (*Oryctolagus cuniculus domesticus*) from three different breeds (one unrelated adult female and male each) were fed with the same pellet food and kept in rabbit cages for three weeks prior to sacrifice. We anesthetized rabbits with intramuscular injection of ketamine (15 mg/kg body weight) and euthanized with intra cardiac injection of sodium pentobarbitone (100 mg/kg body weight). The Ethical Committee for Animal Research of the University of Castilla la Mancha, Spain, approved these experimental procedures with rabbits. One domesticated rabbit (“A115”) showed large expression differences from all other rabbits, explaining almost 40% of gene expression variance in a principal component analysis of all rabbit samples (Figure S7). We excluded this rabbit from all further analyses.

Domesticated guinea pigs (*Cavia porcellus*) and wild cavy (*C. aperea*; six adult animals each, three females each, all unrelated) were housed in same-sex pairs in the same laboratory at the University of Münster, Germany. They were taken from the housing room, immediately anesthetized with isofluran as inhalation anaesthetic (Forene, Abott GmbH, Wiesbaden, Germany) and decapitated. Experiments were announced to the competent local authority (AZ 8.87.50.10.56.08.050) and were approved by the animal protection official of the University of Münster. Guinea pigs were domesticated from the subspecies *C. tschudii* [68]. However, any functional genetic differences that contribute to domestication-specific traits between domesticated guinea pigs and *C. tschudii* are likely to also distinguish them from *C. aperea*.

Tame and aggressive rats (*Rattus norvegicus*; six adult animals each, three females each, all unrelated) derive from a long-running artificial selection experiment at the Institute for Cytology and Genetics in Novosibirsk, Russia. Wild-caught rats had been selected for de- and increased aggression towards humans, respectively, to model the early steps of animal domestication [25]. Rats from the 64th generation of selection had been transferred to the Max Planck Institute for Evolutionary Anthropology in Leipzig, Germany, where behavioral selection had to be stopped because of low fertility. The rats used in this study were from the 5th Leipzig generation. They were taken from the colony room, immediately anesthetized with CO_2_ and killed by cervical dislocation. The rats were part of an animal study approved by the regional government of Saxony (TVV Nr. 29/95).

### RNA–seq data production

Tissues were homogenized in TRIzol reagent (Invitrogen, Darmstadt, Germany), and RNA extracted by chloroform extraction and purified using Quiagen RNEasy columns. All RNA samples were of high and comparable quality as judged by Agilent Bioanalyzer (Agilent Technologies, Böblingen, Germany) “RNA Integrity Values” (Dataset S1). RNA-seq libraries were generated according to standard procedures using Illumina library preparation kits (Illumina Inc., San Diego, USA). Briefly, mRNA was extracted from 10 µg of total RNA by capture on poly-T covered magnetic beads, chemically fragmented and used as template for cDNA synthesis using random hexamer primers. Double-stranded cDNA was blunt-ended and paired-end Illumina sequencing adapters were ligated to the cDNA. Libraries were size-selected around 250 basepairs (bp) by gel extraction from agarose gels and amplified using polymerase chain reaction for 15 cycles. Libraries were sequenced on Illumina GA II instruments (Illumina Inc., San Diego, USA), using one sequencing lane per sample. Paired-end (PE) 51 bp reads were obtained from all samples with the exception of rats, where some samples were 76 bp single-end (SE) or 76 bp PE (Dataset S1). Bases were called using Ibis [79]. To minimize potential experimental batch effects, samples from the respective wild and domesticated species pairs were processed together throughout RNA extraction and library generation, and distributed evenly across sequencing runs.

Raw sequencing reads are available in the ArrayExpress archive (http://www.ebi.ac.uk/arrayexpress/) as accession E-MTAB-1249.

### Read mapping and gene expression quantitation

Illumina reads were processed by trimming adapter sequences, merging overlapping read pairs into single sequences and keeping only reads with ≥51 bp length (after merging and adapter trimming) and ≤5 bases with base call quality ≤15. The remaining reads were mapped to the respective reference genome sequences (CanFam2, Sscrofa9.2, OryCun2, CavPor3 and rn4) using TopHat version 1.0.13 [80]. See Dataset S1 for the number of raw and aligned reads. To avoid potential biases associated with the different run types used in the rats, all rat reads were trimmed to 51 bp SE prior to mapping. We quantified gene expression from the mapped reads using two independent software packages. HTSeq-count (http://www-huber.embl.de/users/anders/HTSeq/doc/count.html) was used to obtain integer counts of mapped reads per gene (using the ‘union’ parameter). Cufflinks version 0.9.0 [81] was used to obtain FPKM [22] expression values, using automatic estimation of the library size distributions and sequence composition bias correction. Read mapping and expression quantification was performed separately for each sample. All calculations using FPKM were performed on *log2(FPKM+1)* transformed expression values. Both programs were run on gene models as defined in the Ensembl 59 database. We did not exclude any genes from the annotations during quantification (*e.g.* paralogues were kept), and did not modify the gene annotations or attempt to identify new genes using our data. Expression levels and differences between samples were not corrected for genetic variation.

Gene expression measures are available as data files for the statistical software R (http://www.r-project.org/) in Dataset S5.

### Differential gene expression in pairs of domesticated and wild species

All statistical analyses were performed in R (http://www.r-project.org/). For each pairwise comparison of wild and domesticated animals, only genes with >0 counts in ≥50% of samples of the given comparison were analyzed (see Table 1 for the number of genes analyzed in each comparison).

In each pair, differentially expressed genes were identified based on integer count data using DESeq version 1.6.1 [45]. Briefly, DESeq determines DE by modeling count data using a negative binomial distribution as follows. First, size factors are calculated that take into account the total number of reads in different samples. Second, for each gene a dispersion parameter is determined that takes into account biological variation between samples. The dispersion parameter is either estimated from a function first fit to all genes that relates the abundance of a gene to its expected dispersion, or directly from the variation observed between replicates. Third, a negative binomial distribution is fit to the counts for each gene. The p-value is calculated as the probability that counts as or more different than observed in the two groups (domesticated or wild) for this gene are obtained under the null distribution that no group difference exists [45]. To ensure conservative significance tests, we used the maximum of the two dispersion estimates per gene, as recommended in the DESeq manual.

To obtain estimates of the variance explained by domestication for a gene *i*, we obtained variance-stabilized expression data from integer counts using DESeq [45] and fitted linear models to the variance-stabilized gene expression levels *y* for each gene *i* using the model specification
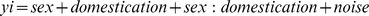
for those species pairs where both sexes were present (dogs, rabbits, guinea pigs, rabbits and rats) and
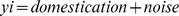
for pigs. The fraction of variance attributable to domestication was calculated as
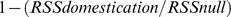
where RSS_domestication_ is the residual sum of squares from the model including domestication, and RSS_null_ is the residual sum of squares from the model without domestication. We assessed significance of these variance estimates by performing all possible permutations of the domestication factor, each time calculating the mean variance explained by the permuted factor.

### Gene Ontology (GO) enrichment analyses

Enrichment analyses for GO (http://www.geneontology.org/) “biological processes” were performed using the R package GOSeq [82], which is able to correct for biases in the power to detect differential expression due to different expression levels. For each gene, the median expression level across all samples was used to control for both transcript length and mRNA abundance differences between genes. GO annotation was extracted from Ensembl version 59 using Biomart. For each gene, Biomart by default only reports the terminal (i.e. most specific) GO terms of the GO graph, potentially leading to a loss of power to detect enrichment in terms higher up in the GO hierarchy. Therefore, custom scripts were used to supplement the GO annotation with all GO terms that are parents of the terms provided by Ensembl. For dogs, pigs, rabbits and rats, genes with a nominally significant (p<0.05) expression difference were tested versus all expressed genes. Due to the large number of DE genes in guinea pigs, we tested the genes with a 10% FDR for differential expression in this species. Dataset S3 lists all GO groups that were significant at a nominal significance level of p<0.01. We did not control for multiple testing in the GO enrichment tests, and the results should therefore be considered exploratory.

### Comparisons between DE genes and maps for positive selection

Genome maps of signals of positive selection during domestication were available for dogs, pigs and rabbits. We used Fisher's exact test (FET) to ask if DE genes in these three species were more likely to show evidence for positive selection than all expressed genes. The contingency tables were constructed by dividing the expressed genes per species into genes that 1) were DE and positively selected, 2) were DE but did not show evidence for selection, 3) were not DE but were positively selected, and 4) were neither DE nor positively selected.

### Analyses of gene expression across species pairs

We tested for overlap among different domestication events using two strategies. The first strategy considers one pair of domestication events at a time, and uses three tests to ask whether there is overlap between them. The second strategy considers all domesticated and wild samples in all species pairs in a joint analyses of variance. The rationale for the second strategy is that smaller shared domestication effects can potentially be uncovered by combining more samples into one statistical analysis.

#### Strategy one—pairwise comparisons

All three tests in this strategy were performed on genes that are expressed (using the above definition) in both domestication events and that are 1∶1 orthologues (as annotated in Ensembl 59) for the given pair of domestication events (*e.g.* keep only 1∶1 orthologues between dogs and pigs). The resulting gene numbers are given in Table S1. First, for each pair of domestication events, one-sided FET was used to ask if DE genes were shared between domestication events. The underlying contingency table for the FET was constructed by dividing the genes into those genes that are 1) DE in both events (*e.g.* differed significantly both between dogs and wolves as well as between pigs and boars), 2) and 3) DE in one event but not the other, and 4) DE in neither event.

The first test is blind to the direction of expression change: a gene that is significantly higher in dogs than in wolves but significantly lower in pigs than in boars would be counted as “shared”. To take into account the direction of expression change, FET was used to test if genes that are DE in one domestication event are more likely to change in the same direction in a second event than all expressed genes. Whether a gene was also DE in the second species was not a criterion for this test. The contingency table for this test was constructed by dividing all 1∶1 orthologues for the given domestication events as follows. The first group comprises genes that are DE in event 1 and have expression change in the same direction in the two events (*i.e.* up in dogs and up in pigs, or down in dogs and down in pigs). The second group comprises genes that are DE in event 1, but have expression change in the second species in the opposite direction (*i.e.* up in dogs but down in pigs or *vice versa*). The third group comprises genes that are not DE in event 1, but changed in the same direction in the two events. Finally, the fourth group comprises the genes that are not DE in event 1, and changed in different directions in the two events. For this test, an odds ratio of one indicates that DE genes in event 1 are as likely to have changed in the same direction in the two events as genes that are not DE in event 1. Odds ratios larger than one indicate that DE genes in event 1 predict the direction of expression change in event 2. Note that this test takes into account overall differences in expression direction, *i.e.* if more genes are more highly expressed in domesticates in both events, the test asks if being DE in event 1 provides additional information about expression direction in event 2. Further note that this test is asymmetric: while DE in event one may predict expression direction in event 2, the inverse is not necessarily true because different genes can be DE in event 2 than in event 1. We therefore conducted this test twice for each pair of domestication events, once with each event serving as “event 1” (e.g. the test was run once using dog DE genes to test for shared direction in pigs, and once using pig DE genes to test for shared direction in dogs). Table 3 and Table 4 report only the result from the more significant of these two test directions.

Finally, the third test for pairwise similarities among pairs of domestication events were Spearman rank correlations between median expression differences across all expressed 1∶1 orthologues, irrespective of whether or not the genes were DE. We assessed significance of the correlations with a permutation test. We randomly permuted the domestication factor 1,000 times in each of the two domestication events, each time calculating Spearman's rho. The permutation p-value reported in Table 4 is the fraction of permutations where the observed statistic is matched or exceeded.

#### Strategy two—analysis of variance

To analyze variance components in the gene expression data, we fitted gene-wise linear models to gene expression data from dogs, wolves, pigs, boars and domesticated and wild rabbits:
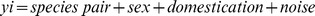



We performed these analyses twice, once using variance-stabilized data [45] based on integer counts obtained from DESeq and once using *log2(FPKM+1)* values. We analyzed only genes that were 1∶1 orthologues in all pairwise comparisons between dogs, pigs and rabbits and that had expression levels >0 in at least half of the samples per species pair in both variance-stabilized and FPKM data. The resulting set comprised 6,901 genes. To assess the significance of the influence of species pair, sex and domestication on gene expression, we performed 1,000 random permutations separately for each factor. The p-value for the influence of a given factor is the fraction of permutations where the median variance explained in the non-permuted data is matched or exceeded. When testing the species pair effect, we randomly assigned each individual to a species. For the sex and domestication effects, we permuted the samples *within* their respective species pair. This strategy was chosen because the differences between species pairs were much larger than the other effects; if domestication and sex effects were permuted randomly across all samples, they would by chance in some permutations be correlated with the species pair factor, which results in artificially high estimates of that factor's effect in the permuted data. The overall consequence would be an anticonservative test of the domestication and sex effects.

### Screen for genes with common expression among domesticated animals

Two criteria were used to identify “domestication-related genes”: 1) a significant main effect for domestication in ANOVA of gene-wise models, and 2) a consistent difference in median expression level (i.e. higher or lower in all domesticated species). We independently searched for genes meeting these criteria using three models. The first two models were the linear models of variance-stabilized and FPKM data described above; for the third model we used the R package edgeR [83] to fit a generalized linear model employing the negative binomial distribution to the integer count data:
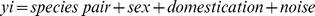
For edgeR, normalization factors were estimated from the data, and gene-wise dispersion parameters estimated using the function estimateCRDisp with a prior.n of 10. Models with and without the domestication terms were fit to each gene, and p-values for the domestication factor obtained from likelihood ratio tests using edgeR's function glmLRT.

For each of these three models, we compared the number of genes that match or exceed a given significance cutoff for the domestication factor to those obtained from all possible “extreme” permutations of the data, where all samples in one species pair completely switch their domestication status (see main text and Note S1 for the rationale behind and a detailed description of this strategy).

### Overlap of putative domestication-related genes with guinea pigs or rats

To test whether the direction of expression change in dogs, pigs and domesticated rabbits is similar to that in the guinea pigs or the tame rats, we performed one-sided FET on genes grouped by their direction of change as described above for pairwise comparisons among domestication events. The observed odds ratios were compared to those obtained from the “extreme” permutations described above. To ensure equally sized gene sets irrespective of the significance cutoff in the extreme permutations, we ranked the genes according to domestication p-value in the edgeR, variance-stabilized and FPKM-based analyses, and selected genes with 1) the highest summed ranks among either or both of these models (*i.e.* the most significant genes), 2) common direction of expression among the domestics in the given permutation, and 3) an expressed orthologue in the target comparison. The number of selected genes was set to match those found in the real data (e.g. 209 genes for comparing guinea pigs to the other pairs).

### Obtaining sequence polymorphism data from RNA–seq data

Given RNA-seq data consists of cDNA sequences, we reasoned that it would be possible to identify single nucleotide variants (SNVs) that segregate in the respective populations, and, where sequence coverage is high, call diploid genotypes for individual samples. To ensure high quality of the genotypes, we applied a series of filters. First, we mapped all reads against the respective genomes using BWA [84], an alignment program that reports mapping quality scores and is able to identify small insertions/deletions (indels) (but is not able to detect spliced reads, making it less ideal for gene expression quantification). Second, to avoid PCR duplicates, we collapsed molecules with identical mapping coordinates, keeping only the read with the highest mapping quality. Third, we trimmed the first and last six bases from each aligned molecule, to avoid sequence errors that can be caused by nonrandom composition of the hexamer pools used during cDNA synthesis, and consequent overwriting of the transcript sequence [85]. Fourth, we kept only alignments with mapping quality >30 and ≤2 mismatches to the reference.

Lists of SNV positions were extracted from merged filtered alignment files from all individuals per species pair (combining domesticated and wild animals), excluding sites with <8 supporting reads, with ≥2 SNVs within a window of 10 bp and within 10 bp of an indel, and sites within repetitive regions (as identified by the repeat masker track from the UCSC genome browser). The individuals were then separately interrogated for their genotypes at these SNV positions. We accepted genotypes produced by samtools [86] if sequence coverage for the given individual was ≥8, consensus quality ≥30, RMS mapping quality ≥25, a maximum of 2 SNVs were present within 10 bp, and the distance to an indel was at least 10 bp. Otherwise, the respective genotype was set to be unknown.

In addition to extracting genotypes at specific SNV positions (referred to as “SNV data”), we also called consensus sequences at all positions in ensembl-annotated exons for each individual, using the same set of filters (referred to as “exome data”).

### Sequence analyses

Only data from sequence positions where all samples in the given comparison had high-quality consensus genotypes were used. Population divergences were calculated from the exome data as the fraction of nucleotides that differ between any two members of the two populations. Table 2 and Figure 2B show the median of these fractions per comparison. Nucleotide diversity (π) was calculated using the program “compute”. Principal component analysis was performed using the R function “prcomp” on SNV data converted to numerical values (e.g. AA = 0; AT = 1; TT = 2).

## Supporting Information

Dataset S1 Full details on samples and sequencing runs performed for this study.
(XLS)

Dataset S2 Differentially expressed genes.
(XLSX)

Dataset S3 GO enrichment results.
(XLS)

Dataset S4 Domestication-related genes.
(XLSX)

Dataset S5 Gene expression values used in the analyses, stored as R objects.
(ZIP)

Figure S1 PCA of gene expression variation. Blue: domesticated/tame rat, red: wild/aggressive rat; circles: females, squares: males.
(PDF)

Figure S2 PCA of SNV data. Blue: domesticated/tame rat, red: wild/aggressive rat; circles: females, squares: males.
(PDF)

Figure S3 Extreme and random permutations when searching for genes with shared expression in domesticated animals. A detailed step-by-step description of this Figure is provided in Note S1. A. Expression levels of a hypothetical gene in three pairs (circles, squares, triangles) of domesticated (blue) and wild (red) animals. The dotted line is the overall mean expression. Left panel: actual data, middle panel: an example of a random permutation of domestication status, right panel: an example of an “extreme” permutation where all members of a given pair have switched their domestication/wild assignment. B. p-value distributions from simulated data with and without domestication effect and with and without random differences within each domesticated/wild pair. The grey diagonal corresponds to a uniform distribution. C. p-value distributions from simulated data with and without random differences, compared to p-value distributions obtained from random and extreme permutations.
(PDF)

Figure S4 Q-Q plots comparing the p-value distribution of the un-permuted data to all possible extreme permutations for the analyses without guinea pigs. p-values are for the effect of domestication in the ANOVA analyses of dogs, pigs and rabbits, and were −log10 transformed. The real, un-permuted data is shown on the y-axis compared to each of the respective extreme permutations on the x-axis. The grey diagonal denotes identity. A: variance stabilized data (vsd), B: FPKM data, C: edgeR analyses of count data.
(PDF)

Figure S5 Transcript sequence differences to the reference genome in genes with common expression in domesticated animals. For each gene, the median fold change of expression in domesticated *vs.* wild animals is plotted as a function of the difference between wild and domesticated mean sequence difference to the reference genome. The p-values are from Wilcoxon rank tests asking if the wild - domestic differences in distance to the reference genome are different between genes with higher vs those with lower expression. Shown are the results for domestication-related genes with significant p-values (p<0.05) in the variance-stabilized, FPKM, and integer count-based models of dogs, pigs and rabbits, and with consistent direction of expression change in guinea pigs.
(PDF)

Figure S6 Q-Q plots comparing the p-value distribution of the un-permuted data to all possible extreme permutations for the analyses including guinea pigs. p-values are for the effect of domestication in the ANOVA analyses of dogs, pigs, rabbits and guinea pigs, and were −log10 transformed. The real, un-permuted data is shown on the respective y-axis compared to each of the respective extreme permutations on the x-axis. The grey diagonal denotes identity. A: variance stabilized data (vsd), B: FPKM data, C: edgeR analyses of count data.
(PDF)

Figure S7 PCA of expression data in all 12 rabbit samples. Note the large distance separating A115 from all other samples. Blue: domesticated, red: wild, circles: females, squares: males.
(PDF)

Note S1 “Extreme” and random permutations when testing for shared gene expression across domestication events.
(DOCX)

Note S2 Sequence polymorphism in genes with common expression in domesticated animals.
(DOCX)

Table S1 Shown is the number of genes that are 1∶1 orthologues in the given comparison and that are “expressed” in both pairs (*i.e.* have >0 counts in at least half the samples in both species pairs that are compared).
(DOCX)
