# Supplementary figures and images for: A Comparison of Brain Gene Expression Levels in Domesticated and Wild Animals
Source: PLoS Genet. 2012 Sep 27;8(9):e1002962. doi: 10.1371/journal.pgen.1002962 (PMC3459979; doi:10.1371/journal.pgen.1002962)

Dog / Wolf

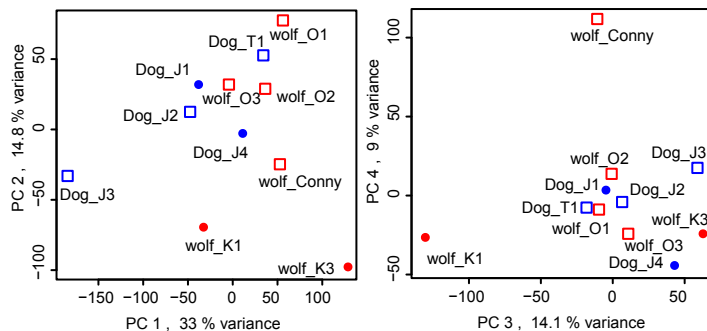

Pig

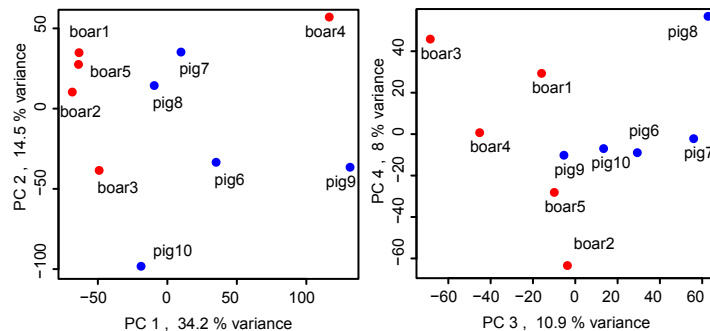

Rabbit

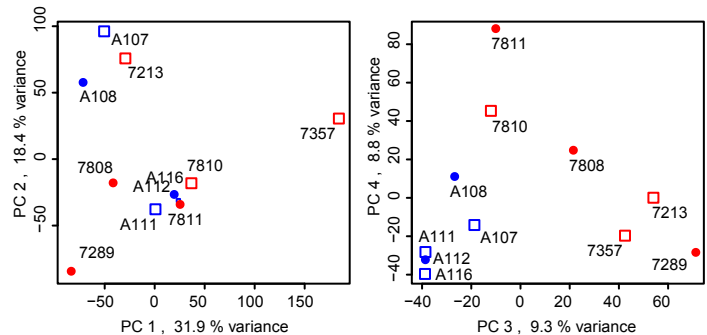

Rat

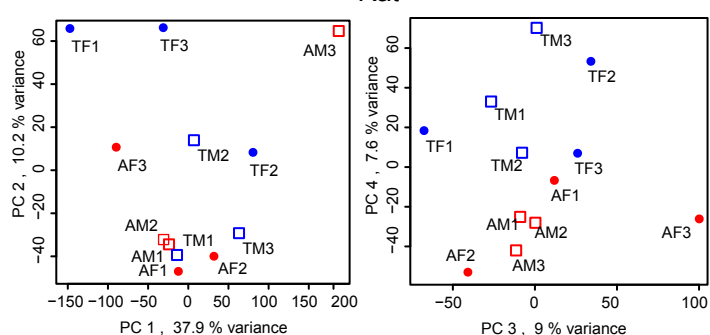

Guinea pig

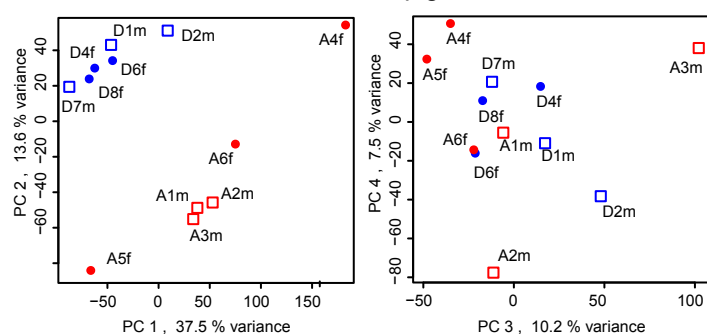

Supplement: Figure S1 — PCA of gene expression variation. Blue: domesticated/tame rat, red: wild/aggressive rat; circles: females, squares: males. (PDF) [file pgen.1002962.s006.pdf]

Dog

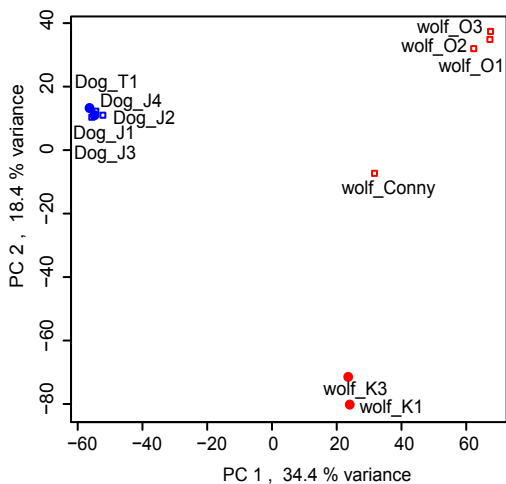

Pig

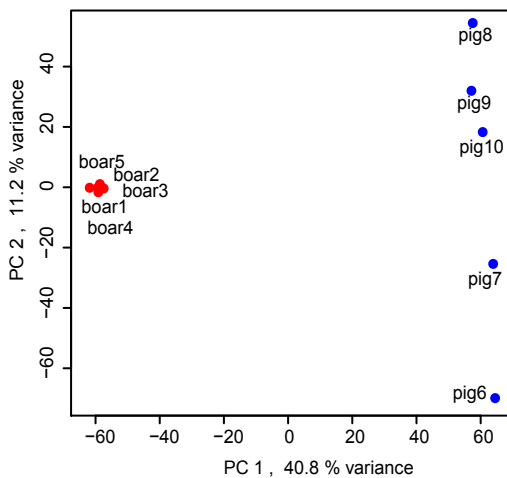

Rabbit

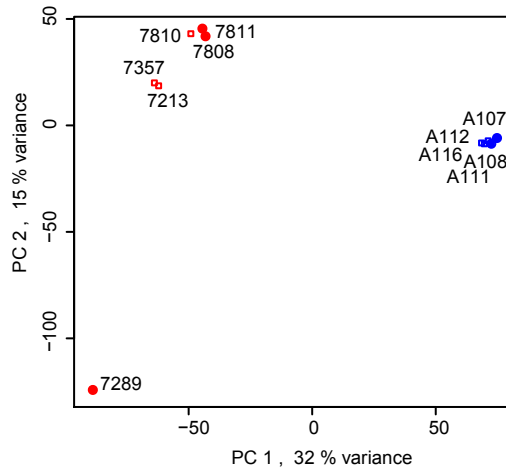

Guinea pig

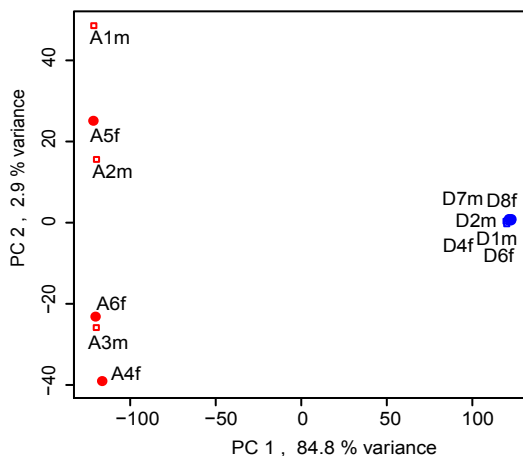

Rat

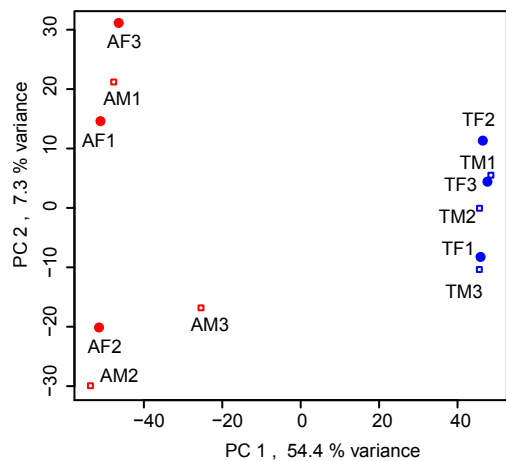

Supplement: Figure S2 — PCA of SNV data. Blue: domesticated/tame rat, red: wild/aggressive rat; circles: females, squares: males. (PDF) [file pgen.1002962.s007.pdf]

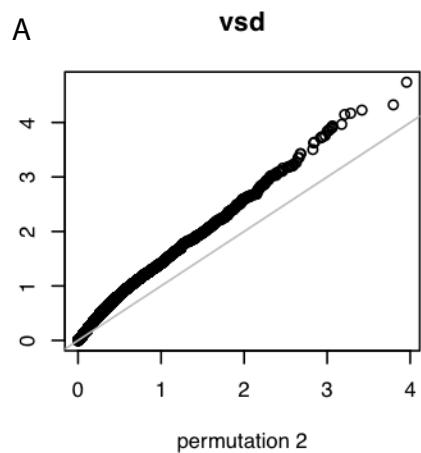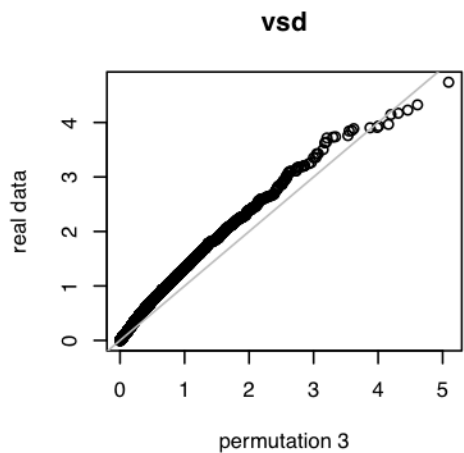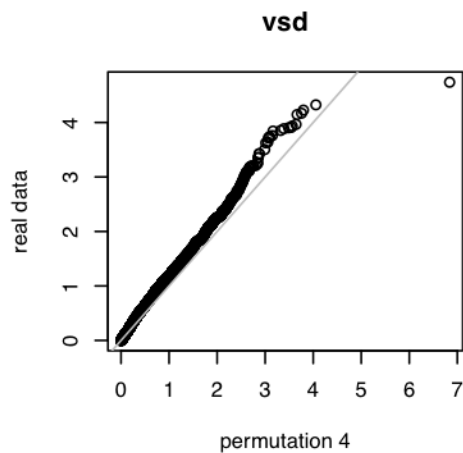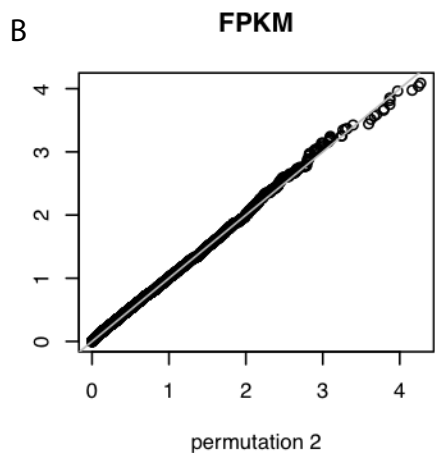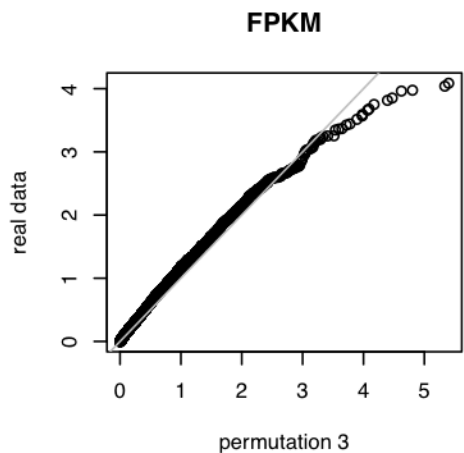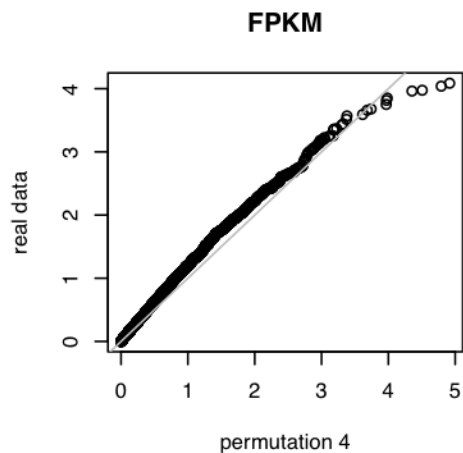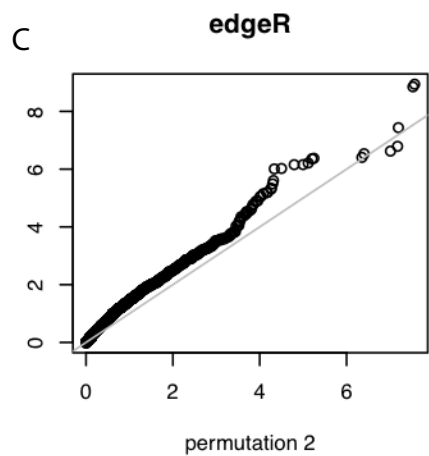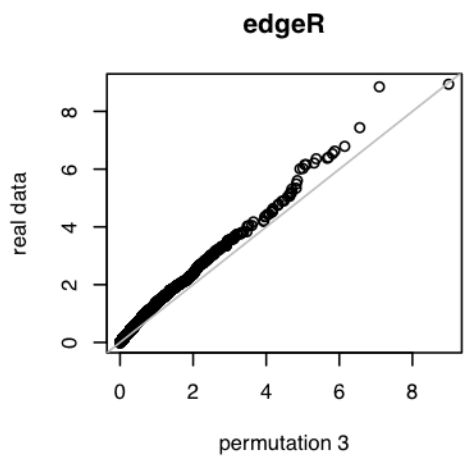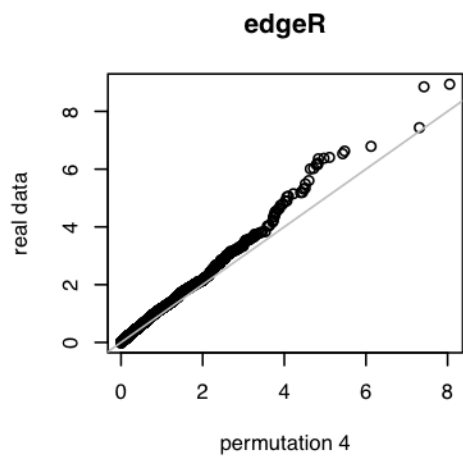

Supplement: Figure S4 — Q-Q plots comparing the p-value distribution of the un-permuted data to all possible extreme permutations for the analyses without guinea pigs. p-values are for the effect of domestication in the ANOVA analyses of dogs, pigs and rabbits, and were −log10 transformed. The real, un-permuted data is shown on the y-axis compared to each of the respective extreme permutations on the x-axis. The grey diagonal denotes identity. A: variance stabilized data (vsd), B: FPKM data, C: edgeR analyses of count data. (PDF) [file pgen.1002962.s009.pdf]

Dog

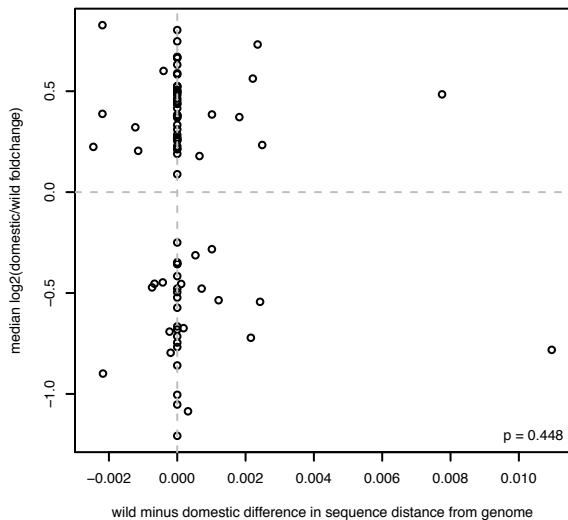

Pig

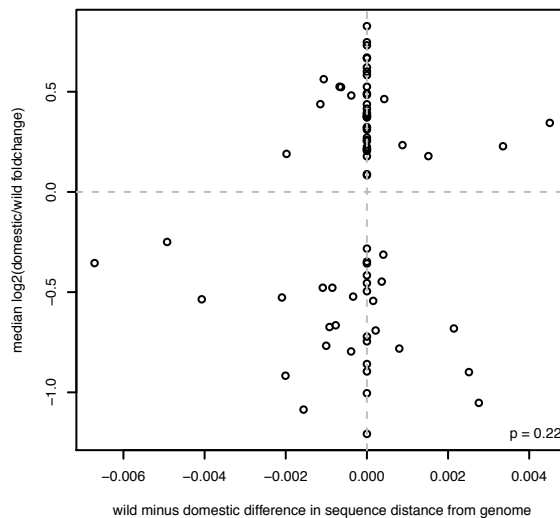

Rabbit

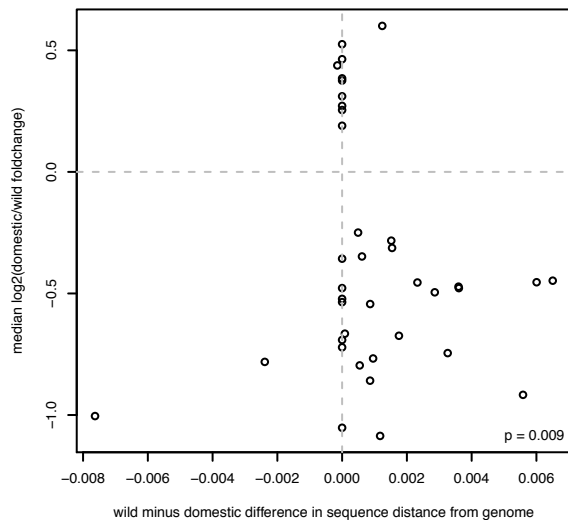

Guinea pig

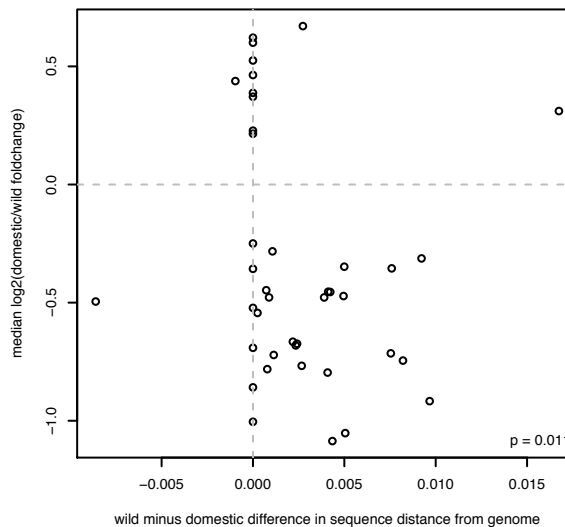

Supplement: Figure S5 — Transcript sequence differences to the reference genome in genes with common expression in domesticated animals. For each gene, the median fold change of expression in domesticated vs. wild animals is plotted as a function of the difference between wild and domesticated mean sequence difference to the reference genome. The p-values are from Wilcoxon rank tests asking if the wild - domestic differences in distance to the reference genome are different between genes with higher vs those with lower expression. Shown are the results for domestication-related genes with significant p-values (p<0.05) in the variance-stabilized, FPKM, and integer count-based models of dogs, pigs and rabbits, and with consistent direction of expression change in guinea pigs. (PDF) [file pgen.1002962.s010.pdf]

A

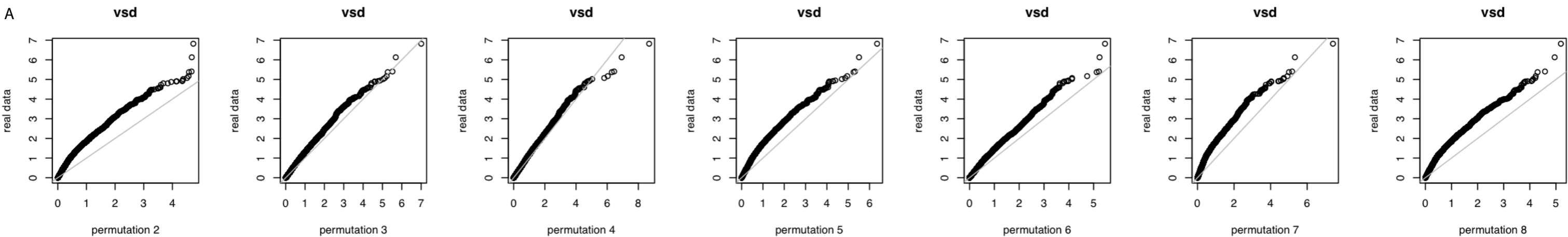

B

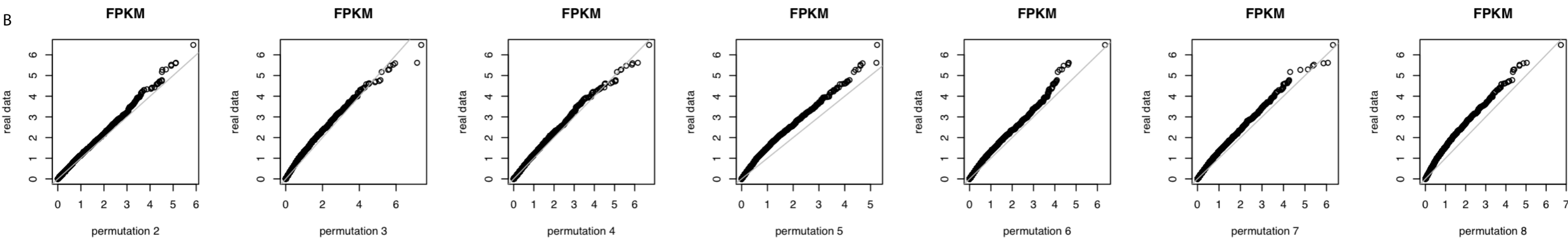

C

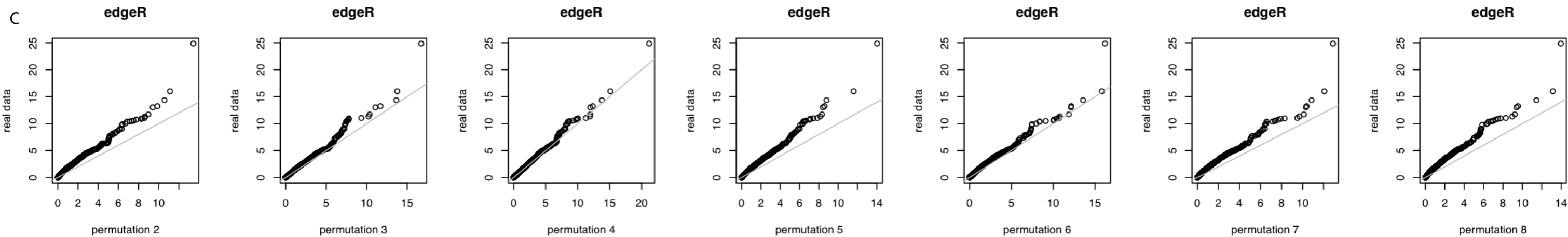

Supplement: Figure S6 — Q-Q plots comparing the p-value distribution of the un-permuted data to all possible extreme permutations for the analyses including guinea pigs. p-values are for the effect of domestication in the ANOVA analyses of dogs, pigs, rabbits and guinea pigs, and were −log10 transformed. The real, un-permuted data is shown on the respective y-axis compared to each of the respective extreme permutations on the x-axis. The grey diagonal denotes identity. A: variance stabilized data (vsd), B: FPKM data, C: edgeR analyses of count data. (PDF) [file pgen.1002962.s011.pdf]

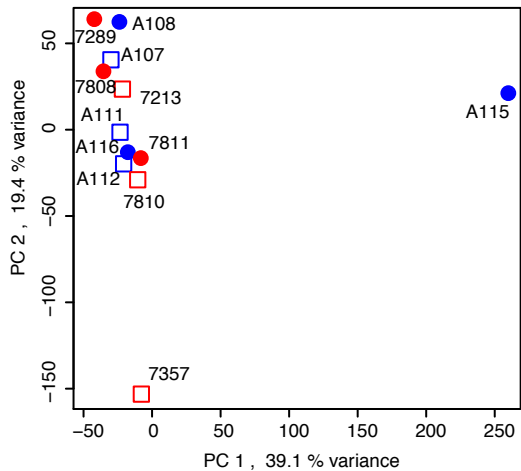

Supplement: Figure S7 — PCA of expression data in all 12 rabbit samples. Note the large distance separating A115 from all other samples. Blue: domesticated, red: wild, circles: females, squares: males. (PDF) [file pgen.1002962.s012.pdf]
