# Supplementary material for: A Comparison of Brain Gene Expression Levels in Domesticated and Wild Animals
Source: PLoS Genet. 2012 Sep 27;8(9):e1002962. doi: 10.1371/journal.pgen.1002962 (PMC3459979; doi:10.1371/journal.pgen.1002962)
Supplement: Figure S3 — Extreme and random permutations when searching for genes with shared expression in domesticated animals. A detailed step-by-step description of this Figure is provided in Note S1. A. Expression levels of a hypothetical gene in three pairs (circles, squares, triangles) of domesticated (blue) and wild (red) animals. The dotted line is the overall mean expression. Left panel: actual data, middle panel: an example of a random permutation of domestication status, right panel: an example of an “extreme” permutation where all members of a given pair have switched their domestication/wild assignment. B. p-value distributions from simulated data with and without domestication effect and with and without random differences within each domesticated/wild pair. The grey diagonal corresponds to a uniform distribution. C. p-value distributions from simulated data with and without random differences, compared to p-value distributions obtained from random and extreme permutations. (PDF) [file pgen.1002962.s008.pdf]

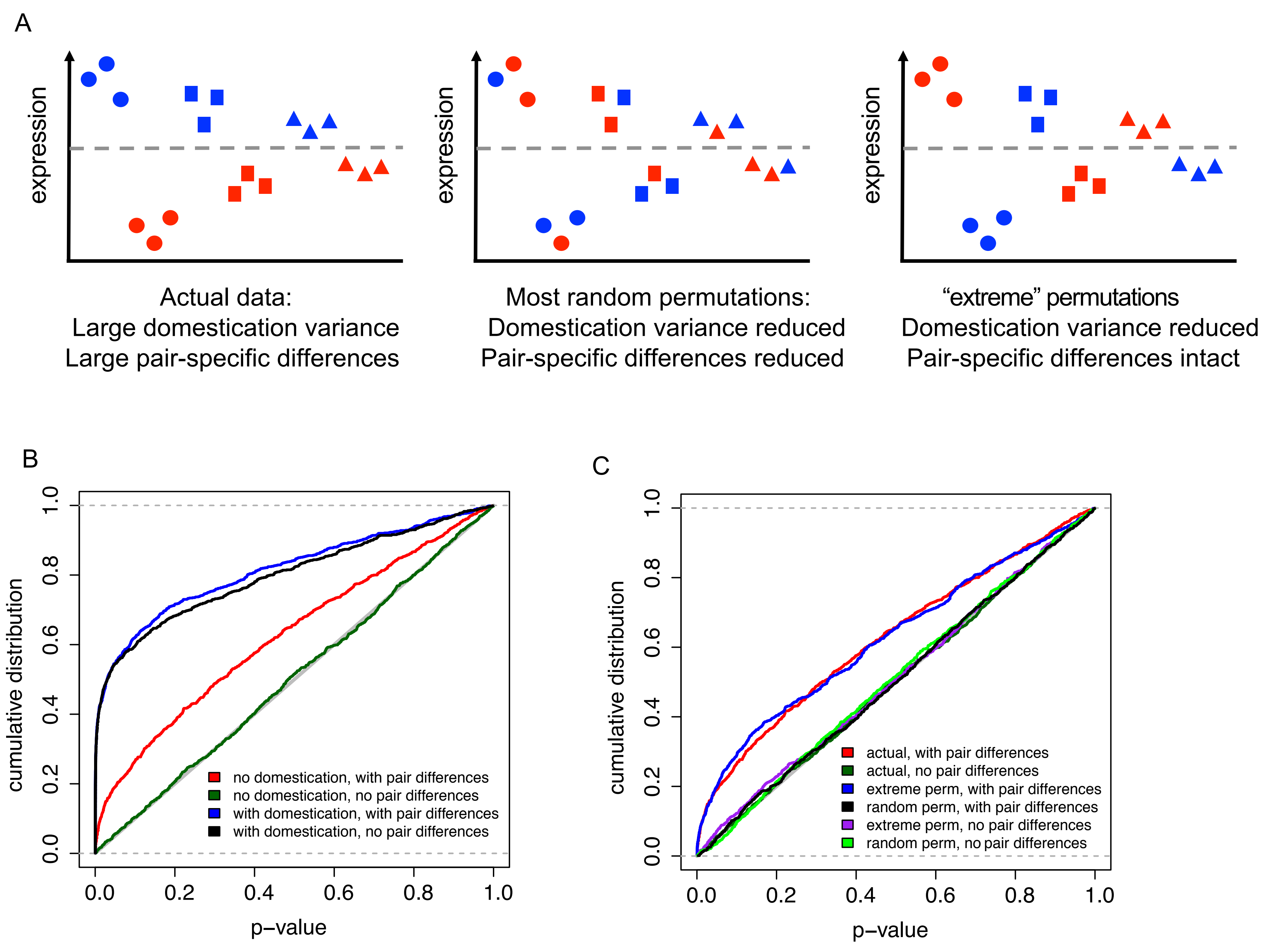
A
expression
Actual data:
Large domestication variance
Large pair-specific differences
Most random permutations:
Domestication variance reduced
Pair-specific differences reduced
"extreme" permutations
Domestication variance reduced
Pair-specific differences intact
B
cumulative distribution
p−value
0.0
0.2
0.4
0.6
0.8
1.0
no domestication, with pair differences
no domestication, no pair differences
with domestication, with pair differences
with domestication, no pair differences
C
actual, with pair differences
actual, no pair differences
extreme perm, with pair differences
random perm, with pair differences
extreme perm, no pair differences
random perm, no pair differences
