# Supplementary material for: A Comparison of Brain Gene Expression Levels in Domesticated and Wild Animals
Source: PLoS Genet. 2012 Sep 27;8(9):e1002962. doi: 10.1371/journal.pgen.1002962 (PMC3459979; doi:10.1371/journal.pgen.1002962)
Supplement: Note S1 — “Extreme” and random permutations when testing for shared gene expression across domestication events. (DOCX) [file pgen.1002962.s013.docx]

Supplementary Note 1 –– “Extreme” and random permutations when testing for shared gene expression across domestication events

In the main text, we point out that with a small number of domesticated / wild species pairs, it is difficult to determine whether an observed p-value for a common domestication effect across species pairs exceeds that expected by chance. We here first illustrate this point with a hypothetical example and then show the existence of the effect with simulations.

Consider Supplementary Figure S3 A. The expression levels of a hypothetical gene are shown for three animals each from three domesticated (blue) and wild (red) species in three species pairs (circles, squares, and triangles). The samples have been normalized so that each species pair has the same overall mean expression, equivalent to including species pair as a factor in our ANOVA analyses (Materials and Methods).

The left panel shows the actual data. Note that each species pair has a difference between domesticated and wild animals, and that they all point in the same direction. Should we be surprised to see a gene with such common differences? It is informative to consider an uninteresting scenario that could lead to the expression pattern in the left panel. Because the domesticated and wild animals within each species pair were separated for some time, they will have accumulated some expression differences, either randomly or because of positive selection. Assume these differences are independent for each of the species pairs so that there is no common process that would, say, cause higher expression in the domesticates. However, because many genes are tested, some genes will show expression change in the same direction in all three species pairs simply by chance. We are interested in determining whether in addition to the chance overlap in direction that will occur for some genes, there is an additional factor (in our case: domestication) that causes systematic changes in gene expression in the three species pairs.

The task is to determine whether the variance explained by domestication for this gene is more (and, equivalently, whether the associated p-value is less) than expected by chance, against a backdrop of thousands of such tests for the other expressed genes. Typically in this situation, permutation tests are performed to determine what distribution of variances / p-values to expect in the absence of real signal. The factor of interest (here: whether an animal is domesticated or not) is permuted many times, the corresponding statistic computed each time, and the distribution of statistics from the real (unpermuted) data compared to the distribution of statistics from the permuted datasets.

However, this strategy fails in the scenario where the dataset is comprised of several species pairs, where the animals within each pair differ from each other by some amount that is uncorrelated from the differences in the other pairs. To see why, consider the middle panel in Figure S3 A, which shows one of many possible random permutations. In this, and in most random permutations, the samples in each species pair are redistributed across domesticated and wild animals. This has two consequences. First the overall variance explained by domestication in this permutation is lower than for the actual data. Second, the differences *within each species pair* are reduced. This second consequence turns out to be crucial. We are not interested in testing whether the within-pair differences are more than expected by chance. We are only interested in whether domestication has led to additional, systematic differences. However, the reduction in domestication variance in this random permutation is at least partly due to the reduced within-pair differences.

The only way to disentangle the contribution of the uninteresting within-pair differences from that of an interesting shared effect of domestication is to use only those permutations that leave the magnitude of the within-pair differences intact while only changing their direction. We termed these “extreme” permutations in the main text because they reassign all domesticated animals per species pair to the wild group and *vice versa*, as illustrated in the right panel of Figure S3 A. In this particular permutation, the variance (p-value) attributable to domestication would be less (higher) than in the actual data, but whether the gene would reach sufficient significance can only be decided by comparing to the distribution of the respective statistics obtained from all genes.

To further illustrate this effect, we simulated a dataset closely mirroring the data presented in this paper (Figure S3 B & C). Expression levels were simulated for three species pairs, each with five wild and five domesticated animals. Expression levels were simulated with large differences among pairs (drawn from a normal distribution with mean = 0 and standard deviation (SD) = 20). Four groups of 1,000 genes were created: with or without random “pair differences” within each pair and with or without a common “domestication” effect. The magnitude of the random pair differences were drawn from a normal distribution closely mirroring the distributions observed in dogs and wolves, pigs and boars, and domesticated and wild rabbits, respectively. Importantly, these pair differences are uncorrelated between the three pairs, *i.e.* the domesticated animals in each pair are shifted by an amount that is independent from the shift in the other two pairs. By contrast, the domestication effect is a *common* shift to all domesticated animals. It was drawn from a normal distribution with mean = 0.5 and SD = 1. Finally, normally distributed residual noise (mean = 0, sd = 1) was added to each individual. The expression level *y* for each gene was analyzed with the linear model

*y ~ pair + domestication + noise*

Figure S3 B and C shows the p-values for the domestication effect obtained from these simulations. Several features are apparent. First, for the case without pair differences and without domestication effect, the p-values are uniformly distributed: they closely follow the diagonal, as expected for a dataset without any signal. However, for the case with random pair differences (the red line in Figure S3 B), the p-values are skewed towards small values, resulting in an apparent excess of significant “domestication p-values“. Note that this occurs without an actual common domestication effect being present – it is purely an artifact of the random pair differences sometimes aligning in the same direction. Finally, when an actual domestication effect is present, there is an excess of significant p-values irrespective of whether random pair differences are present.

Figure S3 C shows the outcome of subjecting the simulated datasets to the “extreme” and the “random” permutation schemes for the case without a common domestication effect. The actual p-value distributions are shown for comparison. First, note that in the case without random pair differences, both permutation schemes produce uniform p-value distributions, as expected given the actual p-values are already uniform. Crucially however, for the case with random pair differences, the random permutation scheme also produces a uniform p-value distribution (black line in Figure S3 C). This is not desirable, as it makes the actual p-value distribution (red) appear shifted towards lower p-values compared to permutations, creating the illusion of an excess of low p-values. To re-iterate, this is the case although no common domestication effect is actually present. By contrast, the “extreme” permutations show the correct behavior (blue line): they closely track the actual p-values. A comparison of the actual p-values to the extreme permutations would therefore result in no apparent excess of low p-values.

We would ideally like to determine, for each of many extreme permutations, how many genes produce p-values smaller than some threshold. The distribution of this number of genes across many permutations can then be compared to the number of genes observed in the actual data. Unfortunately, with a small number of species pairs, not many “extreme” permutations are possible – four (eight) such permutations are possible with three (four) pairs, including the actual data permutation. Rather than the distribution of statistics across many permutations, we therefore present the results from all individual extreme permutations in the main text (e.g. Figure 3 and Supplementary Figures S4 and S5).
