# Supplementary material for: A Comparison of Brain Gene Expression Levels in Domesticated and Wild Animals
Source: PLoS Genet. 2012 Sep 27;8(9):e1002962. doi: 10.1371/journal.pgen.1002962 (PMC3459979; doi:10.1371/journal.pgen.1002962)
Supplement: Note S2 — Sequence polymorphism in genes with common expression in domesticated animals. (DOCX) [file pgen.1002962.s014.docx]

Supplementary Note 2

Sequence polymorphism in genes with common expression in domesticated animals

The reference genomes used to map the RNASeq reads derive from domesticated dogs, pigs, rabbits and guinea pigs, respectively. Therefore, one potential source of bias is sequence polymorphism in the corresponding transcripts. If a gene has more sequence differences to the reference in wild animals than in domesticated animals, its corresponding reads may be more difficult to map to the reference in wild compared to domesticated animals. Fewer mapped reads would lead to lower apparent expression in the wild animals, even if the actual expression level were the same. Therefore, genes with fast evolving transcript sequences may in wild animals have systematically more sequence differences to the reference genomes, leading to consistent, but artifactual expression differences across several species. In particular, those genes with higher expression in the domesticated species could in principle be caused by disproportional transcript sequence divergence in the wild samples.

To test if common expression in domesticated animals is caused by larger sequence distance between the corresponding transcripts and the reference genomes, we used the exome sequences extracted from the RNASeq data. For each gene in each individual, we calculated the fraction of bases that differ from the reference, using only positions where all animals in that species had genotype calls. We then examined for each gene the mean fraction of sequence differences from the reference for the wild and the domesticated animals. Overall, wild animals were more diverged from the reference than domesticated animals (not shown). However, Supplementary Figure S6 shows that these differences were equally distributed among the genes with common expression in domesticated animals. Those genes with higher expression in domestics (top half of the plot) did not have higher sequence divergence in wild animals (right half of the plot) than those with lower expression in domestics. Wilcoxon rank tests asking if the wild - domestic differences in distance to the reference genome are different between genes with higher vs those with lower expression were nonsignificant (p > 0.5) for dogs and pigs. The tests were significant (p > 0.011) for rabbits and guinea pigs, but note that the direction is in the opposite of what could cause the artifact described above: genes with more sequence distance from the reference in wild animals had *lower* expression in wild animals.
