# Supplementary material for: A Comparison of Brain Gene Expression Levels in Domesticated and Wild Animals
Source: PLoS Genet. 2012 Sep 27;8(9):e1002962. doi: 10.1371/journal.pgen.1002962 (PMC3459979; doi:10.1371/journal.pgen.1002962)
Supplement: Table S1 — Shown is the number of genes that are 1∶1 orthologues in the given comparison and that are “expressed” in both pairs (i.e. have >0 counts in at least half the samples in both species pairs that are compared). (DOCX) [file pgen.1002962.s015.docx]

Table S1 – Numbers of genes analyzed in comparisons between domestication events

|  | Dog | Pig | Rabbit | Guinea pig | Rat |
| --- | --- | --- | --- | --- | --- |
| Dog | – |  |  |  |  |
| Pig | 10,296 | – |  |  |  |
| Rabbit | 12,163 | 9,652 | – |  |  |
| Guinea pig | 12,820 | 9,975 | 12,051 | – |  |
| Rat | 12,301 | 9,611 | 11,640 | 12,303 | – |
